# Food and beverage portion sizes in Australian children: a secondary analysis of 1995 and 2007 national data

**DOI:** 10.1186/1471-2458-14-517

**Published:** 2014-05-28

**Authors:** Kate Collins, Jane F Watson, Clare E Collins

**Affiliations:** 1 School of Health Sciences, Faculty of Health and Medicine, University of Newcastle, HA12 Hunter Building, University Drive, Callaghan, NSW 2308, Australia

## Abstract

**Background:**

Portion size of foods is reported to contribute to the rise in obesity prevalence. However, evidence of changes in portion size for commonly consumed foods in Australia is lacking. The aim was to evaluate whether Australian child and adolescent portion sizes of selected foods changed from 1995 to 2007.

**Methods:**

Time-series study, comparing dietary data from two national cross-sectional surveys in nationally representative population survey of Australian households. The dietary data was from children aged 2–16 years who participated in the 1995 National Nutrition Survey (n = 2198) and 2007 Australian National Children’s Nutrition and Physical Activity Survey (n = 4799).

**Results:**

Differences were found across survey years in median portion size of common foods and beverages assessed by 24-hour recalls for age and sex categories. Of the 61 foods items evaluated across the whole population sample, portion size increased in 18 items, decreased in 22, with no change in 20, although the magnitude of change varied by age and sex. Decreases in portion size were detected for most dairy products, breakfast cereal, some packaged snack foods and vegetables, p < 0.0001. Increases were detected for cooked chicken, mixed chicken dishes, bacon and ham (p < 0.0001), cooked meat (p < 0.05), fish (p < 0.01) and pizza (p < 0.0001). No significant changes were detected for many items including white and wholemeal bread, mincemeat, chocolate and soft drink.

**Conclusions:**

Small changes in portion sizes were detected over 12 years in Australian children and adolescents with the degree of change varying by sex, age and food group. Knowledge of usual portion sizes could inform programs targeting appropriate serving sizes selection in children and adolescents.

## Background

The World Health Organization has identified childhood obesity as a serious 21^st^ century public health challenge, with up to 200 million school-aged children classified as overweight or obese [1,2]. Obese children are more likely to remain obese into adulthood and are at an increased risk of developing short and long term health complications such as type II diabetes and cardiovascular disease [3]. In 2011–12 approximately 25% of Australian school-aged children were classified as overweight or obese [4].

An interplay between genetic predisposition and an obesogenic environment has been implicated in causing energy imbalance and leading to the obesity epidemic [5-7]. The physical environment is increasingly conducive to consuming excess energy and a sedentary lifestyle, with children participating in reduced amounts of physical activity and increased amounts of screen time [8]. This has been compounded by a nutritional environment characterized by increased food availability [9], larger portion sizes [10,11], advertising of energy-dense, nutrient-poor foods targeted at children [9], and more people consuming meals away from the home [8]. Furthermore, common dietary behaviors such as the consumption of energy-dense, nutrient-poor foods [6] and high consumption of sweetened beverages [12] have been related to weight gain in children and a compromise in diet quality.

Causal links between portion size and the development of obesity have not been clearly established. Young and Nestle presented the hypothesis that increases in portion size of common foods that occurred over a prolonged period of time contributed to the increased prevalence of overweight and obesity in the USA [13]. Evidence supports the theory that increases in portion size increase overall energy intake [14-18], which in turn can influence weight status of individuals. A positive relationship between body weight, portion size and energy intake (p < 0.05) has been identified in young children (n = 2139) in a US study that examined dietary data [19]. In an adult population, Wansink et al. have explored the role that portion size has on energy intake, influencing consumption norms and reducing an individual’s reliance on self-monitoring [20]. Despite energy intake significantly increasing, adults remained unaware that they had consumed more soup [20] or popcorn [21], and were not more satiated than those who consumed a smaller portion.

Studies quantitatively examining portion size data over time in American and European children are based on routinely collected national nutrition data and conclude that portion size of many energy-dense, nutrient-poor items such as processed snacks [22-24], sweetened beverages [23,25], and take away foods [22,23] have increased over time. Over an 8 year period (1997–2005) in the United Kingdom, portion size of these products increased by 15-20% [24], while over a 21–24 year period (1977-1998/2001) portion size of these items selected by Americans increased 23-60% [22,23,25].

Surprisingly, evidence of changes in portion size for commonly consumed foods in Australia is lacking. The 2007 Australian National Children’s Nutrition and Physical Activity Survey (ANCNPAS) is the most recent national data available that details the eating behaviors of Australian children. Preceding this, dietary data for children of the same age is available from the 1995 National Nutrition Survey (NNS). These national surveys are a primary source of information regarding food consumption patterns and behaviors of Australian children. Previously, techniques have been developed to compare dietary data collected using varying methodologies in the 1995 NNS to the 1985 NNS [26]. While energy intake was shown to significantly increase over this 10 year period, change in portion size was not evaluated [26].

Both the 1995 NNS and the 2007 ANCNPAS utilized a three-pass, face-to-face 24 hour recall as the method of dietary intake data collection from the population [27,28], and hence for the first time provide an opportunity for a directly comparable analysis of portion size within a population of Australian children. Some research has been undertaken utilizing the 1995 NNS and 2007 ANCNPAS dietary data to determine changes in children’s total daily consumption of core and non-core foods [29,30]. This research found that core food consumption had increased across the time period [29] while consumption of energy-dense nutrient poor food had declined, however remained over-consumed providing 35% of energy intake [30]. The current study is the first of this scope comparing portion size per eating occasion across a large number of foods within a sample population of Australian children.

The aim of the current study is to establish whether Australian children’s (2–16 years) portion size of selected foods changed over the 12 year period from 1995 and 2007.

## Methods

### Study design and participants

The time series study compared data from two nationally representative cross-sectional surveys of dietary intake within the Australian population, aged 2–16 years; the 1995 National Nutrition Survey (NNS) (n = 2198) and the 2007 Australian National Children’s Nutrition and Physical Activity Survey (ANCNPAS) (n = 4799). The participants were children and adolescents of the 1995 NNS and 2007 ANCNPAS studies.

This study was approved by the Human Research Ethics Committee of the University of Newcastle, Australia (H-2012-0096).

### 1995 national nutrition survey

The NNS was conducted in a systematic sub-sample of participants from the 1995 National Health Survey [27]. Data collection occurred between February 1995 and March 1996, with a 61.4% response rate [27]. This response rate related to the original sample sizes for the 1995 and 2007 surveys to reflect the numbers of participants in the original dataset. Dietary data was collected through a single three-pass, face-to-face 24-hour recall conducted with a trained interviewer, from 2198 children aged 2–16 years [27]. Parent proxies were reported for children aged two to four years while children aged five to eleven years were asked to report their own food intake data with the assistance of an adult household member [27]. Portion size was estimated using standard household measures, including measuring cups, spoons, rulers, a grid, concentric circles, shapes, containers, and photos of selected food items [27].

### 2007 Australian national children’s nutrition and physical activity survey

The ANCNPAS was conducted using stratified quota sampling by postcode of households with children 2–16 years, and had a 40% response rate [28]. Data was obtained from participants between February 2007 and August 2007 [28]. Dietary data was collected by trained interviewers by two three-pass 24-hour recalls from 4799 children, with the first utilizing computer assisted personal interview (CAPI) and the subsequent utilizing computer assisted telephone interview (CATI) techniques [28]. Data was collected from the primary care giver of 2–8 year olds, and self-reported by children 9–16 years [28]. Portion size was estimated from a food model booklet and measuring aids in CAPI while only utilized a food model booklet in CATI [28]. The food model book contained life-size drawings of containers, plates, bowls, pats of spreads, amorphous mounds, concentric rings, a grid, a moveable wedge, and photographs of common foods such as meat cuts, chocolate bars, drinks, yoghurt, potato chips and snack bars [28].

### Data manipulation

**Figure 1:**
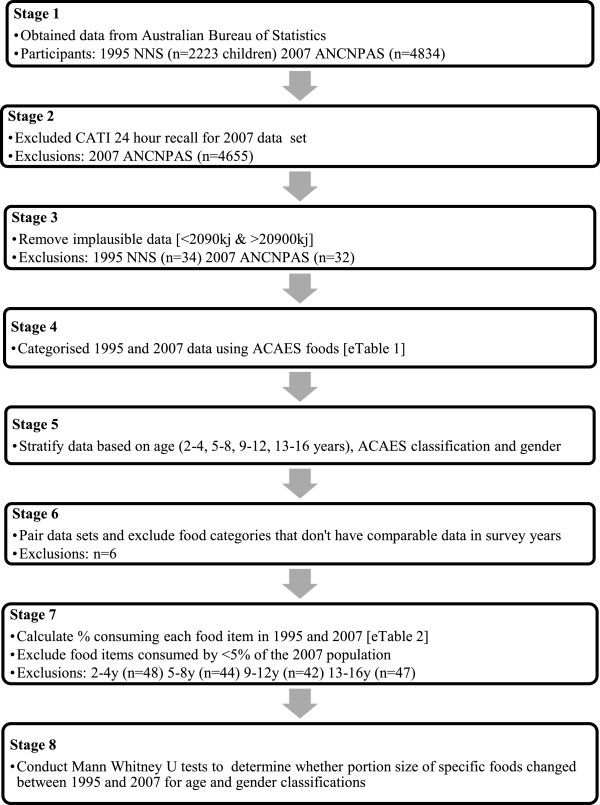
Flow chart describing data manipulation process.

The data manipulation process is detailed in Figure 1. Unpublished portion size data for each survey was obtained from the Australian Bureau of Statistics. Of the two 24-hour recalls collected in the 2007 ANCNPAS only the first (CAPI, 3-pass) was used for analysis to increase the comparability with the 1995 NNS dietary data collected by a three-pass, face-to-face 24 hour recall. The Australian Child and Adolescent Eating Survey (ACAES) Food Frequency Questionnaire [31] was used to categorize foods from each data set for analysis (Additional file 1: Table S1). The food included in the ACAES were derived from focus groups with the target population and designed to include foods that make up the majority of the dietary intakes of Australian children, which was determined by conducting focus groups during the development of the survey [31]. A total of 126 foods were considered for analysis. Implausible intakes from the two national nutrition surveys were excluded based on total daily energy intake using cut-offs from previous research in this age group [32]. In addition, foods were excluded if their consumption rate was less than 5% for the respondents from the 2007 survey population (see Additional file 1: Tables S2 and S3). Cordial was excluded from the analysis due to the inconsistent reporting used in data collection meaning that comparisons could not be made.

### Statistical analysis

Data analysis was conducted by sex and age category (2–4, 5–8, 9–12, and 13–16 years) using JMP 9.0 (2010, SAS Institute Inc., Cary, NC, USA). Differences in demographic characteristics of the 1995 and 2007 participants were assessed through chi-squared or student t-tests. Descriptive statistics were applied to each data set with portion size (grams) of each food expressed as a median. Differences between the portion sizes over time were tested using Mann–Whitney U tests with significance set at p < 0.05.

## Results

Results are based on 6997 children aged 2–16 years who completed the 1995 NNS (n = 2198) or the 2007 ANCNPAS (n = 4799). Table 1 reports demographic characteristics of the samples by year. The 2007 sample mean age was significantly lower in the 2–4 year age group (p < 0.001), and significantly higher in the 5–8 year age group (p = 0.002) compared to 1995. No significant differences existed in mean age of the older groups. There were no significant differences in the proportion of male and female participants between samples. Weight status was determined using international BMI cut-off points [33,34] and significantly differed in the three younger age groups [2–4 years (p = 0.002), 5–8 years (p < 0.001), 9–12 years (p = 0.03)], with a higher percentage of the 2007 sample being of a normal weight. Children surveyed in 2007 reported a lower mean daily energy intake (8330kj/day) than their 1995 (8621kj/day) counterparts (p < 0.001) and this was consistent when stratified by sex [30]. In each of the age groups analysed, the 1995 sample had a significantly greater proportion reporting their previous day’s dietary intake as unusual.

**Table 1:** Demographic data of participants of the 1995 NNS and 2007 ANCNPAS

	**2-4 years**		**5-8 years**		**9-12 years**		**13-16 years**	
	**1995**	**2007**	**1995**	**2007**	**1995**	**2007**	**1995**	**2007**
**n =**	581	1491	624	954	544	966	449	1388
**Mean age (y) [SD]**	2.97^c^ [0.81]	2.83^c^ [0.74]	6.42^b^ [1.12]	6.60^b^ [1.12]	10.47 [1.14]	10.53 [1.10]	14.47 [1.12]	14.55 [1.01]
**Sex (%)**								
Male	49	52	55	51	53	47	52	50
Female	51	48	45	49	47	53	48	50
**Weight status**^**† **^**(%)**								
Underweight	8.1	4.4	4.5	3.7	2.9	5.6	4.5	4.1
Normal	70.7	74.6	63.9	76.2	65.8	67.0	67.5	70.4
Overweight	13.1	15.4	23.6	13.6	23.7	19.4	20.5	17.8
Obese	5.0	3.4	7.7	6.2	7.4	7.2	6.0	6.4
Unknown	3.1	2.1^b*^	0.3	0.3^c^*	0.2	0.8^a^*	1.6	0.4
**Intake (%)**								
Usual	65.7	97.9	63	97.1	61.4	72.5	56.6	72.3
Unusual	32.7	2.1^c^*	36.4	2.9^c^*	38.2	27.5^c^*	43.2	27.7^c^*

^a^p value <0.05.

^b^p value <0.01.

^c^p value <0.001.

*The distribution of the specified categorical variables significantly differs between 1995 and 2007.

^†^When decimal age could not be determined it was assumed age was midway through the year [43,44].

Only foods that were consumed by at least five percent of the population were considered in the analysis (see Additional file 1: Tables S2 and S3). The total number of foods analysed for each age group, after excluding foods consumed by less than 5 percent of the population, were as follows: 65 foods analysed for 13–16 year olds, 71 foods for 9–12 year olds, 66 foods for 5–8 year olds, and 65 foods for 2–4 year olds (see Additional file 1: Tables S4-S7).

Table 2 reports all foods for which a significant change in portion size was detected from 1995 to 2007. Irrespective of sex, portion size changed significantly in 39% (n = 104) of foods studied, with 15% (n = 40) increasing in size, and a further 24% (n = 64) of foods decreasing in portion size. The remaining 61% of foods had no significant change in portion size.

**Table 2:** Significant changes in median portion size (grams) of commonly consumed foods between 1995 and 2007

**Food**	**2-4 years**			**5-8 years**			**9-12 years**			**13-16 years**			**2-16 years**
	**Female**	**Male**	**Persons**	**Female**	**Male**	**Persons**	**Female**	**Male**	**Persons**	**Female**	**Male**	**Persons**	**Persons**
**Drinks**													
Soft drink	-45^b^	-48^c^	-44^c^	-	-	-	-50^a^	-	-23^a^	-	-	-	-
Water	-320^c^	-300^c^	-312^c^	-270^c^	-250^c^	-250^c^	-450^c^	-500^c^	-500^c^	-475^c^	-450^c^	-450^c^	-250^d^
Fruit juice	-62^c^	-62^c^	-62^c^	0^↓b^	-	0^↓a^	0^↓c^	-	-21^b^	-1^a^	-	-1^a^	-3^d^
Tea/Coffee	-	-	-	-	-	-54^a^	-14^c^	-14^b^	-14^b^	-14^c^	-	-14^b^	-14^d^
**Dairy foods**													
Whole milk	-31^b^	-29^c^	-39^c^	-	-	-17^b^	-	-1^b^	-21^a^	77^a^	-	0^↑a^	-12.9^d^
Reduced fat milk	0^↓a^	-125^c^	-65^c^	-39^a^	-66^c^	-25^c^	-1^a^	-23^a^	-53^b^	-	-	-	-14.5^d^
Skim milk	-	-	-	-	-	-	-	-22^a^	-65^b^	-	-	-	-73.3^d^
Flavored milk	-67^a^	-	-45^a^	-	-	-8^b^	-	-	-	-	-	-	-24^d^
Ice cream	-13^b^	-14^c^	-15^c^	-37^c^	-40^c^	-36^c^	-	-34^a^	-	-	-66^b^	-16^b^	-21^a^
Yoghurt cheese	-25^a^	-	-	-	-42^a^	-50^a^	-107^c^	-100^b^	-100^c^	-	-	-	-50^d^
	-	1^a^	0^↑a^	1^b^	1^a^	1^c^	1^a^	-	0^↑b^	1^a^	9^b^	2^c^	1 ^d^
Cheese spread	-9^b^	-8^c^	-8^c^	-	-	-	-	-	-	-	-	-	-8^d^
**Breads and cereals**													
White bread	-11^b^	-12^b^	-9^c^	-	-	-	-	0^↓a^	-	-	-	-	-
Wholemeal Bread	-	-	-	-	-	-	-	-	-	10^a^	-	10^a^	-
Mixed grain bread	-	-	-	-	22^c^	12^a^	-	-	-	12^a^	0^↑a^	2^a^	8^b^
Breakfast cereal	-	-	-4^a^	-	-	-	-	-15^a^	-	-	-	-	-6.5^d^
Hot porridge	-	-65^b^	-70^c^	-	-	-	-	-	-	-	-	-	-135^d^
English muffins	-	-	-	-	-	-	-	-	-	-	34^b^	7^b^	5^a^
Rice	-	-	-	-	-	-	-32^a^	-	0^↓a^	-	-	-	-46^b^
**Sweets and snacks**													
Cake	-5^a^	-	-	-	-	-	-	-	-	9^a^	-	-	-
Sweet pastries	-	-	-	-	-	-	-23^a^	-	-	-	-	-	-
Sweet biscuits	-	-	-	6^b^	-	4^b^	-	-	-	-	-	-	-
Chocolate/Cream biscuits	-	-	-	-	-	-	9^c^	-	3^a^	-	-7^a^	-10^b^	-
Muesli bars	-	-	-	-	0^↑a^	0^↑a^	-	-	-	-	-	-	-
**Main meals**													
Mince	-	-	-	-	-	-	-	-	42^a^	-58^a^	-	-	-
Fresh cooked chicken	-	-	-	-	-	5^a^	-	-	16^a^	34^a^	-		8.5^d^
Mixed chicken dish	-	-	-	-	-	-	-	-	-98^a^	-	-	-	125^d^
Crumbed chicken	-	-	-	-	-	-	-	58^a^	37^a^	-	58^c^	28^b^	18^b^
Fresh cooked meat	-	-	-	-	-	-	28^a^	15^b^	23^c^	-	-	-	8^a^
Fish	23^c^	22^a^	23^c^	11^a^	24^b^	13^c^	-	-	-	-	-	-	37.1^b^
Sausages	-	-	-	0^↓a^	-	-1^b^	-	-	-	-	-	-	-1^b^
Pizza	-	-	-	44^a^	-	-	-	47^a^	-	69^c^	125^c^	83^c^	50^d^
**Packaged snacks & other foods**													
Chips (not potato)	-	-4^a^	-3^b^	-	-	-	16^c^	-	16^c^	-	-	-	-
Potato crisps	-5^a^	-4^a^	-4^c^	-4^c^	-	-4^c^	-4^b^	-4^a^	-4^c^	-4^a^	-	-4^b^	-4^d^
Chocolate	-3^a^	-5^a^	-4^b^	-	-	-	-	-	-	-	-	-10^a^	-
Tomato sauce	-	-	-	-	5^b^	3^b^	2^a^	-	-	11^c^	0^↑a^	9^c^	2^d^
Lollies	-	-	-	-	-	-	2^a^	-	1^a^	-	-	-	1^c^
Ice block (creamy)	-	-	-	-	-	-	-9^b^	-	-10^b^	-	-	-	-12.5^d^
Jam/Honey	-	-	-	-	-	-	5^a^	-	3^a^	6^b^	-	3^b^	4.0^d^
Peanut butter	-	-	-	-	-	-	2^b^	8^c^	3^c^	-	-	-	2.5^a^
Luncheon meats	10^a^	-	-	11^b^	-	10^a^	17^a^	7^a^	13^c^	-	-	-	-
Bacon/Ham	5^b^	7^a^	7^c^	9^c^	7^a^	6^c^	3^b^	4^a^	4^c^	5^a^	-	3^a^	2.3^d^
Egg	-	-	-	-	-	-	-	-	-	-	-24^a^	-	-7^b^
**Fruit & vegetables**													
Hot chips	-	-15^b^	-15^b^	-25^c^	-23^b^	-25^c^	-	-40^c^	-24^c^	-	-	-	0
Potato	-	-	-2^a^	-41^a^	-	-18^b^	-16^c^	-17^a^	-14^c^	-	-	-	-30^d^
Green beans	-	-	-	-	-	-	-	-	-	-	-21^a^	-	-
Peas	-14^a^	-8^a^	-12^c^	-20^a^	-18^b^	-20^c^	-26^b^	-19^a^	-20^c^	-	-33^a^	-12^a^	-16.8^d^
Broccoli	-	-21^b^	-	-	-19^a^	-	-35^a^	-	-2^a^	-	-	-18^a^	-15.8^d^
Carrot	-	-	-	-6^a^	-	-	-	-10^a^	-	-	-	-	-0.2^c^
Cauliflower	-	-	-	-	-	-	-	17^a^	-	-	-	-	9^a^
Corn	-	-	-	-	-	-17^a^	-	-	-	-	-	-	-
Capsicum	-	-	-	-	-	-	-	8^a^	7^a^	-	-	-	-
Eggplant/Zucchini	-	12^a^	-	-	-	-	-	-	-	-	-	-	-
Tomato	-	-	-	-	-	-	-	-	-	15^b^	-	12^b^	7.5^b^
Lettuce	-	-2^a^	-	-	-	-	-	-	-	2^c^	-	1^a^	-
Onion	-	-	-	-	-	-	-	-8^b^	-4^b^	-	-11^b^	-9^c^	-6. 2^d^
Canned fruit	-	-	-50^a^	-	-	-	-	-	-	-	-	-	-55^c^
Apple	-	-	-	-	-	21^a^	-	-	-	26^b^	26^c^	26^c^	6^a^
Orange	-	-	-	-	-45^a^	-15^a^	-30^a^	-	-15^a^	-	-	-32^a^	-15^c^
Banana	17^a^	4^a^	11^b^	25^c^	25^c^	25^c^	-	-	19^a^	15^b^	19^b^	19^c^	5^d^
**Seasonal fruit**													
Berries/Grapes	-	-27^a^	-	-	-	-	-	-	-	-	-	-	-
Melon	-	-174^a^	-75^a^	-	-	-	-	-	-	-	-	-	-

Positive values imply that portion size increased across the time period whereas negative values imply a reduction in portion size of the given food occurred.

- Indicates no significant change in portion size occurred.

^a^p value <0.05.

^b^p value <0.01.

^c^p value <0.001.

^d^p value <0.0001.

0^↑^ Change in median portion size was 0, however the Mann Whitney U test found a significant increase in portion size distributions in 2007, when compared to 1995.

0^↓^ Change in median portion size was 0, however the Mann Whitney U test found a significant reduction in portion size distributions in 2007, when compared to 1995.

Sex was not related to the changes in portion size, with a similar numbers of significant changes found for both females (n = 79) and males (n = 75). In relation to age, the 9–12 year age group had a greater number of significant changes in portion size than other age groups. Children aged between two and twelve years had more significant reductions in portion size of beverages and dairy foods.

Over the 12 year period, reductions in portion size of packaged foods were detected across all sex and age categories. Of note were potato crisps and yoghurt, which decreased by 16-52% over at least six of the population sub-groups analysed. In contrast, the portion size of luncheon meats and main meal dishes, with the exception of sausage and mixed poultry, increased significantly within numerous population sub-groups.

The portion size of water decreased by 250-500 mL across all groups. Reductions also occurred for soft drink, fruit juice and tea/coffee.

## Discussion

This study evaluated whether portion size of selected foods had changed within a nationally representative sample of Australian children between 1995 and 2007. During this same time period, the prevalence of obesity in children increased from 5% to 8%, while the prevalence of overweight remain fairly constant over time (17%) [4]. Over the same 12 year period, changes to portion size were evident in some foods, although many did not change.This suggests that portion size per se is not related to weight status. The current analysis found that portion size of many energy-dense, nutrient-poor foods remained stable or reduced across the 12 year study period. Previous research examining consumption patterns of energy-dense, nutrient-poor ‘extra’ foods, and core foods using the same data set found that children’s consumption of non-core foods had reduced, while core food consumption had increased [29,30]. This suggests that these improvements in dietary patterns may be partly attributed to changes in portion sizes detected in the current analysis. Portion size of many packaged foods sold through supermarkets has reduced over time [35]. This may be in response to recommendations in the Australian Guide to Healthy Eating (AGHE), released in 1998, stating a serving of energy-dense, nutrient-poor, ‘extra’ food should provide no more than 600kj per serve [36]. The current study suggests that a variety of packaged foods such as soft drink, flavoured milk, creamy ice blocks, yoghurt, potato chips and chocolate had decreased in portion size in selected age and sex groups. With the exception of flavoured milk, the percentage of children consuming these foods remained stable or decreased over time. Regardless, consumption rates remain high (Supplementary Additional file 1: Tables S2 and S3) and hence foods such as soft drink, ice cream, creamy ice blocks, potato chips and chocolate, which are energy dense and of a lower nutrient density, continue to significantly contribute to daily energy intake of Australian children.

With the exception of cheese, reductions in portion size of dairy based foods occurred across all age groups. These were paralleled by reductions in the percentage of children consuming most dairy foods, excluding flavoured milk. This may explain why 44-89% of older children surveyed in 2007 failed to meet their Estimated Average Requirement for calcium [37]. These findings are consistent with international literature. Nielsen et al. found that over a 24 year period the portion size and consumption of milk based beverages had reduced in American children [25].

This is the first paper internationally to analyse portion size changes in a large variety of meat-based foods and main meals in a population of children. It was found that portion size of the majority of meat-based foods, with the exception of sausages in the 5–8 year group, remained stable or increased between 1995 and 2007. Median portion size of fresh cooked meat, poultry and seafood however, remained within AGHE recommendations of 65-100 g of cooked meat [36]. The World Cancer Research Fund recommends total avoidance of processed meats [38]. Portion size of processed meats increased across all age groups, a concerning finding given the convincing links to colorectal cancer [38].

Consumption of fruit and vegetables has been associated with the prevention of cardiovascular disease, overweight and obesity, and some types of cancers [39]. Additionally, eating larger portions of these foods with a meal reduces overall energy consumption [40,41]. This study found portion size of very few vegetables increased over the 12 year period. With the exception of potato products and pumpkin, the median portion size of vegetables (see Additional file 1: Tables S4-S7) did not meet the serve size of 75 g per serve specified in the AGHE [36]. Mean vegetable consumption per capita in 2007 within children aged 2–16 years was 117 g (1.56 serves) [29], well below recommendations of 4–5 serves [36]. Based on the 2007 portion size data children would need to consume 7–10 portions of vegetables each day to meet the total amount recommended in the AGHE.

Conversely, portion size of commonly consumed fruits such as apples and bananas increased, while the percentage of children consuming these foods increased or remained stable. This increase in portion size of fruit that are usually consumed as discrete pieces may be partly attributed to the size of individual pieces commonly sold in supermarkets. With the exception of apples in children aged 5–16 years, portion size of fruit met only 50-80% of the serving size of 150 g specified in the AGHE [36]. This suggests children need to consume 3–4 portions of fruit each day to meet recommendations.

When interpreting these results a number of limitations must be considered. Limitations regarding national survey data have been previously described [29,30] and include changes to seasonality and the age of self-report for dietary data. Further limitations surround the reporting of food items. Each item recorded for the 24-hour recalls was considered a single portion size and these portions were not altered for the present study. That is, a single portion was the amount of each food consumed during one eating occasion. Detailed methodology for the collection of the 24-hour recall data within the National Nutrition Survey has been reported by the Australian Bureau of Statistics previously [27,37]. Misclassification of mixed foods such as hamburgers and burritos/tacos may have occurred if foods were reported as their constituent ingredients. Beverages also appeared to be commonly misreported, with implausibly high intakes leading to large variations in portion size. As a result cordial was excluded from statistical analysis, and changes detected for water should be interpreted with caution. Due to the similarities in sampling frame for the 1995 and 2007 surveys, unweighted data is reported consistent with previous national reports [26,42]. The results are presented in gender and age groups but were not adjusted for geographic variation in portion size estimates and therefore this remains in these data comparisons. Finally, assumptions made about participants were subject to limitation. Although an upper limit of 20900kj has been used in various studies [31,32] as a cut off for over reporting, it is plausible that some 13–16 year olds could require more than 20900kj to meet energy requirements. Furthermore weight status for participants in the 1995 NNS was determined using mid-age as decimal age was not available. This approach was consistent with other literature [43,44].

Strengths of this study include that it is the first of its kind to analyse changes in portion size for a national sample of Australian children. The use of 24-hour recalls allowed reliable estimates of group intake [45] and the methods in which dietary data was collected from participants were consistent between time points, making results obtained directly comparable.

The prevention of childhood obesity in Australia has been at the forefront of public health messages in recent years. Government and non-government bodies have developed initiatives including school canteen guidelines, media campaigns, food advertising restriction, school-based nutrition and physical activity programs, and targeted community interventions. Reductions in the size of many commercially available packaged foods are likely to have contributed to reductions in the portion sizes consumed by children and potentially contributed to changes in body weight at the population level. Changes in health status and nutrition-related behaviors, including portion size selection noted in this analysis may be somewhat attributed to these efforts.

Additionally, the Australian Government established the Food and Health Dialogue in 2009 in response to the results of the 2007 ACNPAS [46]. The Dialogue is a collaboration between the Australian Government, food industry (including food manufacturers, retailers and foodservice operators) and public health groups. It is a voluntary program aiming to facilitate changes in the food supply, including reduced portion sizes of foods, to enable better access to healthier food choices for all Australians. Given the public health implications, it is recommended that adherence to the Food and Health Dialogue Food Category Action Plans is mandated for reductions in food portion sizes. To be effective, any information relating to portion size should be understandable, usable and acceptable to consumers [47].

Furthermore, an emphasis should be placed on children consuming a variety of vegetables as a key strategy to increase intake. The current study suggests this may be 7–10 smaller portions of vegetables to achieve the total amount of vegetables recommended in the AGHE.

Monitoring of dietary intake is necessary to direct future health promotion initiatives and public policy. It is recommended that national monitoring of dietary intake of children is continued using consistent data collection methods to ensure comparable results.

In conclusion, changes in portion size between 1995 and 2007 for Australian children have been variable. Portion size of soft drink, ice cream, creamy ice blocks, potato chips and chocolate, dairy foods, beverages and vegetables has reduced, while the size of meat-based dishes and fruits have increased. These findings have implications for future dietary recommendations and policy related to standard portion sizes.

## Abbreviations

ANCNPAS: Australian National Children’s Nutrition and Physical Activity Survey; NNS: National Nutrition Survey; CAPI: Computer Assisted Personal Interview; CATI: Computer Assisted Telephone Interview; ACAES: Australian Child and Adolescent Eating Survey; AGHE: Australian Guide to Healthy Eating.

## Competing interests

The authors declare that they have no competing interests.

## Authors’ contributions

CEC initiated the concept, KC conducted the analyses with assistance from JFW. KC conducted this work as part requirement for the degree Bachelor of Nutrition and Dietetics (Honours) in the School of Health Sciences, The University of Newcastle, Australia. KC drafted the initial manuscript, all authors interpreted the results, read, critically revised, and approved the final manuscript.

## Authors’ information

CEC, PhD, BSc, Dip Nutr&Diet, Dip Clin Epi, Advanced Accredited Practising Dietitian, Dietitians Association of Australia (APD), Fellow of the Dietitians Association of Australia (FDAA), Professor of Nutrition and Dietetics, School of Health Sciences, Faculty of Health and Medicine, University of Newcastle, NSW, Australia, KC (Honours Candidate), Bachelor of Nutrition and Dietetics, University of Newcastle, NSW, Australia.JFW, PhD BHSc (Nutr&Diet), APD, School of Health Sciences, Faculty of Health and Medicine, University of Newcastle, NSW, Australia.

## Pre-publication history

The pre-publication history for this paper can be accessed here:

http://www.biomedcentral.com/1471-2458/14/517/prepub

## Supplementary Material

Additional file 1: Table S1 NNS and ANCNPAS Food Classification. **Table S2.** Percentage Consumption of Selected Foods in Children aged 2-4 years and 5-8 years. **Table S3.** Percentage Consumption of Selected Foods in Children aged 9–12 years and 13–16 years. **Table S4.** Changes to portion size (grams) between 1995 and 2007 for 2–4 year old children. **Table S5.** Changes to portion size (grams) between 1995 and 2007 for 5–8 year old children. **Table S6.** Changes to portion size (grams) between 1995 and 2007 for 9–12 year old children. **Table S7.** Changes to portion size (grams) between 1995 and 2007 for 13–16 year old children. Click here for file
