# Supplementary material for: Food and beverage portion sizes in Australian children: a secondary analysis of 1995 and 2007 national data
Source: BMC Public Health. 2014 May 28;14:517. doi: 10.1186/1471-2458-14-517 (PMC4061520; doi:10.1186/1471-2458-14-517)
Supplement: Additional file 1: Table S1 — NNS and ANCNPAS Food Classification. Table S2. Percentage Consumption of Selected Foods in Children aged 2-4 years and 5-8 years. Table S3. Percentage Consumption of Selected Foods in Children aged 9–12 years and 13–16 years. Table S4. Changes to portion size (grams) between 1995 and 2007 for 2–4 year old children. Table S5. Changes to portion size (grams) between 1995 and 2007 for 5–8 year old children. Table S6. Changes to portion size (grams) between 1995 and 2007 for 9–12 year old children. Table S7. Changes to portion size (grams) between 1995 and 2007 for 13–16 year old children. [file 1471-2458-14-517-S1.pdf]

Additional files – Tables S1 to S7

Table S1 NNS and ANCNPAS Food Classification P2

Table S2 Percentage Consumption of Selected Foods in Children aged 2-4 years and 5-8 years P13

Table S3 Percentage Consumption of Selected Foods in Children aged 9-12 years and 13-16 years P23

Table S4 Changes to portion size between 1995 and 2007 for 2-4 year old children P33

Table S5 Changes to portion size between 1995 and 2007 for 5-8 year old children P37

Table S6 Changes to portion size between 1995 and 2007 for 9-12 year old children P41

Table S7 Changes to portion size between 1995 and 2007 for 13-16 year old children P45

Table S1: NNS and ANCNPAS Food Classification

Question	FFQ Item	NNS (1995) Foods	ANCNPAS (2007) Foods
[redacted]			
D1	DIET soft drink eg. Diet coke	Soft drinks, artificially sweetened	Soft drink, intense sweetened
D2	Soft drink (NOT DIET) including flavoured mineral water eg. lemonade, coke, fanta, flavoured mineral water	Soft drink,<br>Water, mineral, fruit flavoured	Water, mineral, with 5% fruit<br>Water, mineral, fruit flavours<br>Soft drink
D3	Water - including bottled water. unflavoured mineral water, tap water	Water, …	Water, tap<br>Water, sports type<br>Water, rainwater<br>Water, mineral, natural<br>Water, carbonated or soda<br>Water, bottled..<br>Water, bore
D4	Fruit juice-based drinks eg. orange juice or Popper	Single fruit juices	Juice concentrate,<br>Juice,<br>Vegetable blend –Juice Plus
D5	Cordial or 'make up' eg. Cottee's crush, raspberry	Fruit-based or flavoured cordials and drinks	Cordial,<br>Drink, fruit flavoured,<br>Fruit drink<br>Fruit puree drink,
D6	Tea or Coffee	Tea, coffee or coffee substitutes	Coffee mix,<br>Coffee,<br>Tea,
D7	Beer	Beers	Beer, lager<br>Beer, reduced alcohol or light style
D8	Wine or wine coolers eg. West Coast cooler	Wine, ….	Wine
D9	Spirits eg. vodka, bourbon	Brandy, …<br>Whiskey, …<br>Gin, …<br>Rum, …<br>Vodka, …<br>Spirit, …<br>Liqueur, …<br>Mixed Drink, ..	Alcoholic Soda<br>Liqueur<br>Rum, dark & light coloured<br>Whisky<br>Vodka
[redacted]			
DF1 + DF3	Soy Milk	Milk - soy (unflavoured)	Soy beverage, unflavoured
DF1 + DF3	Whole Milk	Milk - fat increased/whole milk	Milk, cow, fluid, extra creamy<br>Milk, cow, fluid, regular fat
DF1 + DF3	Reduced Fat Milk	Milk - fat reduced	Milk, cow, fluid reduced fat -1-2%-<br>Milk, cow, fluid, reduced fat
DF1 + DF3	Skim Milk	Milk, fluid, skim, non-fat	Milk, cow, fluid, skim

Table S1: NNS and ANCNPAS Food Classification (cont)

Question	FFQ Item	NNS (1995) Foods	ANCNPAS (2007) Foods
DF2	Flavoured milk eg. Moove, Oak, hot chocolate, milkshake, thickshake, smoothie	Flavoured milk, …	Beverage, banana or similar flavour<br>Beverage, caramel, honeycomb or strawberry flavour<br>Beverage, chocolate flavour<br>Beverage, drinking chocolate<br>Beverage, from unfortified base<br>Beverage, malt flavour<br>Milk, cow, fluid, flavoured<br>Milkshake
DF4	Cream or sour cream	Cream, …..	Cream, sour<br>Cream, whipped
DF5	Ice cream - vanilla, chocolate, strawberry, sundaes, cones	ice cream -vanilla rich/regular fat<br>ice cream -other flavour regular fat/rich<br>ice cream -reduced fat<br>ice cream –other	Ice cream sundae<br>Ice cream, reduced fat<br>Ice cream, regular fat<br>Ice cream, soft serve
DF6	Frozen yoghurt	Frozen yoghurts, all types	Yoghurt, frozen
DF7	Yoghurt (not frozen) plain or flavoured eg. Ski, Yoplait, Vaalia	Yoghurt, …	Yoghurt, (except frozen)
DF8	Cottage cheese or ricotta	Cheese, cottage<br>Cheese, ricotta	Cheese, cottage<br>Cheese, ricotta..
DF9	Cheese including cheese on sandwiches, biscuits or on toast	Total other cheese	Cheese, blended<br>Cheese, cheddar<br>Cheese, Colby<br>Cheese, Feta
DF10	Cheese spread, cream cheese	Cheese, cream<br>Cheese, spread	Cheese spread,<br>Cheese, cream<br>Cheese, spread
B1	Muesli	Muesli, …	Muesli, commercial,<br>Muesli, homemade
B2	Cooked porridge	Breakfast cereal, hot porridge type	Oats,<br>Porridge,
B3	Breakfast cereal eg. Weet-bix, Nutri-grain, Cornflakes Sultana Bran	Breakfast cereal, low added sugar, puffed, flakes, extruded etc. single cereal — corn, rice<br>Breakfast cereal, wheat-based biscuits and shredded wheat<br>Breakfast cereal, wheat, low added sugar, puffed, flakes<br>Breakfast cereal, wheat, with added fruit or nuts<br>Breakfast cereal, grain mixtures<br>Breakfast cereal, grain and fruit/nut mixtures<br>Breakfast cereal, other sweetened	Breakfast Cereal,

Table S1: NNS and ANCNPAS Food Classification (cont)

Question	FFQ Item	NNS (1995) Foods	ANCNPAS (2007) Foods
B5	Bread, pita bread, roll or toast all types – White	Bread, white<br>Bread roll, white	Bread, from white flour<br>Bread roll, from white flour<br>Bread or bread roll, from white flour
B5	Bread, pita bread, roll or toast all types – Mixed Grain	Bread, mixed grain<br>Bread roll, mixed grain<br>Bread, light rye/black/pumpernickel	Bread, mixed grain<br>Bread roll, mixed grain<br>Bread or bread roll, mixed grain
B5	Bread, pita bread, roll or toast all types - Wholemeal	Bread, wholemeal<br>Bread roll, wholemeal	Bread, from wholemeal flour<br>Bread roll, from wholemeal flour<br>Bread or bread roll, from wholemeal flour
B6	English muffin, bagel or crumpet	English muffins/crumpets and bagels	Bagel<br>Crumpet,<br>Muffin, English-style,<br>Muffin, savoury,
B7	Rice	Rice and rice products	Rice, brown<br>Rice, flavoured<br>Rice, purchased as<br>Rice, white
B8	Other grains eg. cous cous, burghul		Cous cous,<br>Cornmeal –polenta<br>Millet, puffed
B9	Noodles eg. egg noodles (yellow), rice noodles (white)	Noodles, Asian style	Noodle,
B10	Pasta eg. spaghetti, lasagne, pasta bake	Pasta and egg noodles/filled pasta, ravioli	Gnocchi, potato, boiled<br>Macaroni cheese,<br>Pasta or noodles, buckwheat flour<br>Pasta, … filled<br>Pasta, white<br>Pasta, wholemeal
[redacted]			
S1	Cakes, sweet muffins, scones, pikelets, pancakes, hot cakes eg. apple muffin, chocolate cake, lamington	Cake,….<br>Bun,….<br>Muffin,…..<br>Scone,…..<br>Batter-based product,….	Brownie, chocolate<br>Cake,<br>Cookies,<br>Doughnut<br>Hotcake,<br>Muffin, cake or American style<br>Pancake, plain<br>Pikelet or drop scone,<br>Scone,

Table S1: NNS and ANCNPAS Food Classification (cont)

Question	FFQ Item	NNS (1995) Foods	ANCNPAS (2007) Foods
S2	Sweet pies or sweet pastries eg. apple pie, Danish	Sweet pastry, ….<br>Tart, ….<br>Slice, ….	Baklava<br>Bun or scroll,<br>Croissant,<br>Danish style pastry,<br>Dumpling, sweet<br>Éclair, chocolate<br>Pie, apple<br>Pie, apricot<br>Pie, lemon<br>Pie, pumpkin<br>Pie, pecan<br>Pie, fruit -<br>Tart,<br>Slice,
S3	Other puddings or desserts (not ice cream)<br>eg. chocolate mousse, sticky date pudding	Custards, and sweet sauces, milk-based<br>Dairy desserts, smooth or gelatin-based dairy desserts<br>Sugar-based desserts, …	Crumble,<br>Custard, dairy,<br>Custard, egg,<br>Dairy or yoghurt dessert,<br>Dessert, Bavarian cream,<br>Fudge,<br>Mousse,<br>Pudding,<br>Rum balls<br>Trifle, homemade<br>Souffle, berry
S4	Plain sweet biscuits eg. Arrowroot, Morning Coffee, Tiny Teddies	Biscuit, sweet, plain …<br>Biscuit, sweet, fruit filled ….	Biscuits, shortbread style<br>Biscuits, sugar-glazed pastry<br>Biscuits, sweet, Anzac<br>Biscuits, sweet, chocolate chip<br>Biscuits, sweet, dreid fruit filling<br>Biscuits sweet, ginger flavoured<br>Biscuits, sweet, iced<br>Biscuits, sweet, jam filled<br>Biscuits, sweet, macaroon<br>Biscuit, sweet, oatmeal<br>Biscuit, sweet, plain<br>Biscuit, sweet, sponge finger<br>Biscuit, sweet, wheatmeal<br>Biscuit, sweet, with dried fruit<br>Biscuit, sweet, with nuts
S5	Cream or chocolate biscuits eg. Tim Tams, shortbread cream	Sweet biscuits, cream-filled<br>Sweet biscuits, chocolate-coated, chocolate chip	Biscuits, sweet, chocolate coated<br>Biscuits, sweet, chocolate flavour.<br>Biscuits, sweet, chocolate topped<br>Biscuit, sweet, cream filled
S6	Dry or savoury biscuits, crispbread, crackers eg. Vita Weats, Saos, Jatz, Shapes, rice crackers, Cruskits	Biscuit, savoury	Biscuit, savoury cake<br>Biscuit, savoury cracker<br>Biscuit, savoury crispbread<br>Biscuit, savoury<br>Biscuit, sweet, vanilla

Table S1: NNS and ANCNPAS Food Classification (cont)

Question	FFQ Item	NNS (1995) Foods	ANCNPAS (2007) Foods
S7	Savoury combination snacks - biscuits and cheese eg. Le Snak, Snack abouts	-	Savoury snack, biscuit and cheese, …
S8	Sweet combination snacks eg. Dunkaroos	-	-
S9	Snack noodles eg. 2-minute noodles, Monster noodles	Snack, noodle-based	Noodle, wheat, instant
S10	Fruit bars eg. Roll Ups	Fruit bar and fruit-based confectionery	Fruit leather
S11	Snack bars eg. K-time twist bar	-	Bar, fruit filled<br>Bar, snack
S12	Muesli bars eg. Yoghurt Tops	Bar, muesli…	Bar, breakfast and snack style, cereal<br>Bar, muesli
[redacted]			
M1	Mince dish eg. spaghetti bolognese, rissoles, shepherd's pie, lasagne		Beef, mince<br>Cannelloni,, beef<br>Lamb, mince<br>Lasagne,<br>Pasta bake, beef<br>Pasta Bolognese
M2<br>M3<br>M5<br>M11<br>M12	Beef or lamb pieces and sauce WITHOUT vegetables eg. beef stroganoff<br>Beef or lamb pieces and *sauce* WITH vegetables eg. stir fry<br>Plain meat (beef or lamb) (eg. roast, chops, steak) WITH vegetables or salad<br>Pork pieces and *sauce* WITHOUT vegetables eg. sweet and sour pork<br>Pork pieces and *sauce* WITH vegetables eg. stir fry	Mixed dishes where beef or veal is the major component<br>Mixed dishes where lamb or pork, bacon, ham is the major component	Beef, curry<br>Beef, stirfry<br>Beef, casserole or stew<br>Lamb, curry<br>Lamb, stewed or casseroled<br>Pork, stirfry<br>Pork, sweet and sour

Table S1: NNS and ANCNPAS Food Classification (cont)

Question	FFQ Item	NNS (1995) Foods	ANCNPAS (2007) Foods
M4	Plain meat (beef or lamb) (eg. roast, chops, steak) WITHOUT vegetables or salad	Beef<br>Lamb<br>Veal	Beef, (except mince)<br>Lamb,<br>Veal,
M6<br><br>M7<br><br>M10	Chicken pieces and *sauce* WITHOUT vegetables eg. satay chicken<br>Chicken pieces and *sauce* WITH vegetables eg. stir fry<br>Plain chicken (eg. roast or BBQ) WITH vegetables	Mixed dishes where poultry or game is the major component	Chicken, salad<br>Chicken, stew or casserole<br>Chicken, stir fry<br>Chicken, curry
M8	Chicken crumbed eg. chicken nuggets, KFC pieces, schnitzel	Chicken, crumbed ….	Chicken, nugget<br>Chicken, various pieces<br>Chicken,….., crumbed
M9	Plain chicken (eg. roast or BBQ) WITHOUT vegetables	Chicken<br>Other poultry	Chicken, breast<br>Chicken, baked<br>Chicken, cooked<br>Chicken, drumstick<br>Chicken, grilled.<br>Chicken, kebab<br>Chicken, thigh<br>Chicken, whole<br>Chicken, wing
M13	Plain pork (eg. roast or chops) WITHOUT vegetables	Pork	Pork
M15	Liver – beef, calf, chicken (including paté)	Organ meats and offal, products and dishes	Chicken, liver<br>Pate

Table S1: NNS and ANCNPAS Food Classification (cont)

Question	FFQ Item	NNS (1995) Foods	ANCNPAS (2007) Foods
M16 + M17	Fish	Barramundi, …<br>Blackfish, …<br>Blue Grenadier, …<br>Bream, …<br>Cod, …<br>Trout, …<br>Dory, …<br>Flathead, …<br>Flounder, …<br>Kingfish, …<br>Leather jacket, …<br>Mullet, ..<br>Mackerel, …<br>Perch, …<br>Salmon, …<br>Shark, ….<br>Snapper, …<br>Tailor, …<br>Trevally, …<br>Tuna, …<br>Whiting, …<br>Fish, …	Bassa –basa-,<br>Blue grenadier – hoki -,<br>Bream<br>Fish finger, …<br>Fish,<br>Flathead,<br>Mullet, yelloweye,<br>Whiting,<br>Trout<br>Trevally, dory, ling, cod, flounder or sole<br>Snapper,<br>Shark-flake- skinless fillet,<br>Sliver perch,<br>Barramundi<br>Cod,<br>Mulloway,<br>Salmon,<br>Tuna, yellowfin steaks
M18	Canned tuna, salmon, sardines including patties	Anchovy, canned …..<br>Herring, canned ….<br>Mackeral, canned …<br>Salmon, …, canned …<br>Sardine, canned …<br>Tuna, canned …..	Anchovy, canned<br>Salmon, canned<br>Tuna, canned,<br>Tuna, flavoured, canned<br>Sardine, canned<br>Fish cake
M19	Other seafood eg. prawns, lobster	Crab, …<br>Lobster, ..<br>Bug, …<br>Prawn, …<br>Yabbie, …<br>Abalone, …<br>Calamari, …<br>Clam, …<br>Mussel, …<br>Oyster, …<br>Scallop, …	Crab, various types,<br>Crabmeat,<br>Lobster,<br>Mussel, green,<br>Oyster, baked or grilled<br>Oyster, raw<br>Prawn,<br>Squid or calamari,<br>Scallop,
M20<br>M21	Creamy soup<br>Clear soup with rice or noodles	Soup, …	Soup, …
M22	Tacos, burritos, enchiladas	Taco and tortilla-based dishes	Burrito,<br>Enchilada,<br>Taco,
M23	Sausages, frankfurts, Pluto Pup	Sausage<br>Frankfurts, and saveloys	Frankfurt or cheerios,<br>Sausage,
M24	Hamburger - all types	Chickenburger<br>Fishburger<br>Hamburger	Chickenburger<br>Fishburger,<br>Hamburger,
M25	Pizza	Pizza	Pizza,

Table S1: NNS and ANCNPAS Food Classification (cont)

Question	FFQ Item	NNS (1995) Foods	ANCNPAS (2007) Foods
M26	Pie, sausage roll, chiko roll	Chiko roll<br>Meat pie<br>Sausage roll	Chiko roll, deep fired<br>Pie, meat.<br>Pie, steak<br>Sausage roll,
M27	Hot dog	Hot dog	-
M28	Savoury pastries eg, spinach and cheese triangles	Other savoury pastries	Cannelloni, spinach and ricotta<br>Curry puff, beef,<br>Dim sim, meat and vegetable filling<br>Dumpling, meat filled<br>Dumpling, prawn filled<br>Pastie, vegetable<br>Pastry, cheese filling.<br>Pastry, spinach and cheese filling .<br>Pastry, spring roll<br>Pastry, vegetable filling
M29	Hash browns, potato scallops	Hash Brown,<br>Potato, gems<br>Potato, scallops	Potato, gems<br>Potato, hash brown<br>Potato, mashed & crumbed<br>Potato, scallop
O1	Chips (not potato) eg. Twisties, corn chips, burger rings	Corn snacks and extruded snacks	Corn chips<br>Crisp or chip, soy<br>Crisp or chip, vegetable,<br>Crisp, savoury
O2	Potato chips or crisps eg. plain, salt and vinegar	Potato crisps	Crisp or chip, potato<br>Potato straws
O3	Ice block – creamy eg. Paddle Pop, Magnum, Cornetto	Ice cream, individual bar, stick and cone varieties	Ice cream bar<br>Ice cream, stick
O4	Ice block – water eg. Frosty Fruit, lemonade	Water ice confection, gelato	Gelato, various flavours, commercial<br>Ice confection, stick
O5	Chocolate eg. plain chocolate, Mars Bar, Snickers, Milky Way	Chocolate and chocolate-based confectionery	Chocolate or chocolate bar<br>Chocolate<br>Rocky Road<br>Turkish delight
O6	Lollies without chocolate eg. lollipops, snakes, Skittles, Starburst	Other confectionery	Caramels<br>Honeycomb, plain<br>Liquorice<br>Marshmallow<br>Sugar confectionary
O7	Low fat salad dressing or mayonnaise	Mayonnaise and salad dressing - low fat	Dressing, reduced fat<br>Mayonnaise, low fat
O8	Salad dressing or mayonnaise - not low fat	Mayonnaise and salad dressing - full fat	Dressing, regular<br>Mayonnaise, full fat
O9	Nuts eg. peanuts, almonds	Nuts (excluding coconut)	Nut<br>Nuts, mixed

Table S1: NNS and ANCNPAS Food Classification (cont)

Question	FFQ Item	NNS (1995) Foods	ANCNPAS (2007) Foods
O10	Jam, honey, golden syrup, marmalade	Honey, jam, lemon butter	Honey<br>Jam<br>Marmalade<br>Syrup, maple.<br>Syrup, golden
O11	Peanut butter, Nutella	Peanut butter, chocolate and hazelnut spread	Peanut butter, smooth & crunchy<br>Spread, hazelnut & chocolate flavoured
O12	Vegemite, Mighty Mite, Promite, Marmite	-	Spread, yeast
O13	Tomato sauce, barbecue sauce	Sauce, tomato<br>Sauce, barbecue	Sauce, tomato<br>Sauce, barbecue
O14	Devon, salami	Salami,<br>Chicken roll,<br>Stassburg,<br>Luncheon meats	Kabana or cabanossi<br>Salami
O15	Bacon, ham	Bacon<br>Ham	Bacon<br>Ham
O16	Eggs eg. boiled, scrambled	Egg	Egg
O17	Jelly	Jelly, made up	Jelly, made up
F1<br><br>F2	Hot chips bought from a shop (eg. McDonald's fries) or cooked at home	Potato - hot chips	Potato, chips<br>Potato, fries<br>Potato, wedged
F3	Potato boiled, mashed, baked	Potato - boiled, mashed, baked	Potato, baked<br>Potato, boiled<br>Potato, mashed,<br>Potato, peeled,<br>Potato, unpeeled
F4	Pumpkin	Pumpkin	Pumpkin, peeled
F5	Sweet potato	Sweet potato	Sweet potato,
F6	Cauliflower	Cauliflower	Cauliflower,
F7	Green beans	Beans - green and snake	Bean, green<br>Bean, broad<br>Beans, not further specified
F8	Spinach	Spinach and silverbeet	Mixed greens<br>Spinach<br>Silverbeet
F9	Cabbage or brussel sprouts	Cabbage and brussel sprouts	Brussels sprout<br>Cabbage
F10	Peas	Peas (including snow peas)	Pea, green<br>Pea, split<br>Snow pea
F11	Broccoli	Broccoli	Broccoli
F12	Carrots	Carrots	Carrot

Table S1: NNS and ANCNPAS Food Classification (cont)

Question	FFQ Item	NNS (1995) Foods	ANCNPAS (2007) Foods
F13	Zucchini, eggplant, squash	Squash<br>Zucchini<br>Eggplant	Eggplant<br>Okra<br>Zucchini<br>Squash
F14	Capsicum	Capsicum	Capsicum
F15	Corn, sweetcorn, corn on the cob	Corn	Sweetcorn
F16	Mushrooms	Mushrooms	Mushroom
F17	Tomatoes	Tomatoes (includes canned and sun dried)	Tomato, common
F18	Lettuce	Lettuce	Lettuce, raw
F19	Celery, cucumber	Celery<br>Cucumber	Celery Cucumber
F20	Avocado	Avocados	Avocado, raw
F21	Onion, spring onion, leek	Onions, leeks, spring onions and shallots	Onion, mature<br>Onion, spring Shallot<br>Leek
F22	Soybeans, tofu	Soy beans<br>Tofu	Bean, soya<br>Tofu –soy bean curd
F23	Baked beans	Baked beans	Baked beans
F24	Other beans, lentils eg. chickpeas, split peas	Other mature legumes and pulses<br>Other legume and pulse products<br>Other Beans - broad, butter, kidney	Bean, black<br>Bean, butter<br>Bean, lima<br>Bean, mixed<br>Bean, red kidney<br>Beans, refried<br>Chickpea<br>Lentil
F25	Canned fruit eg. peaches, Two fruits	Fruit - canned, mixed<br>Fruit - canned single	Apple, canned in syrup<br>Apricot, canned<br>Peach, canned<br>Pear, canned<br>Fruit, canned
F26	Fruit salad	Fruit salad (not canned)	Fruit salad
F27	Dried fruit eg. sultanas, dried apricots	Fruit NFS, dried<br>Apple, dried<br>Apricot, dried<br>Banana, chip<br>Berries, dried<br>Date, dried<br>Fig, dried<br>Mixed fruit, dried<br>Prune<br>Sultana	Apple, dried<br>Apricot, dried<br>Banana chip<br>Blueberry, dried<br>Berries, mixed -strawberry, raspberry, blueberry, blackberry-, dried<br>Cranberry, dried<br>Currant, dried<br>Date, dried<br>Fig, dried<br>Mango, dried<br>Mixed fruit, dried<br>Pineapple, dried<br>Prune –dried plum<br>Sultana, dried

Table S1: NNS and ANCNPAS Food Classification (cont)

Question	FFQ Item	NNS (1995) Foods	ANCNPAS (2007) Foods
F28	Apple or pear	Apple, ….<br>Pear, ….	Apple, green skin<br>Apple, peeled, raw<br>Apple, red skin<br>Pear, raw
F29	Orange, mandarin, grapefruit	Oranges,…<br>Mandarin, ….<br>Grapefruit, ….	Mandarin<br>Grapefruit<br>Orange
F30	Banana	Bananas	Banana, cavendish, peeled, raw
FS1	Peach, nectarine, plum or apricot	Apricot<br>Nectarine<br>Peach<br>Plum	Apricot, fresh, raw<br>Nectarine, unpeeled, raw<br>Peach, fresh, raw<br>Plum
FS2	Mango or paw-paw	Mango, peeled<br>Pawpaw	Mango, peeled<br>Pawpaw
FS3	Pineapple	Pineapples	Pineapple, fresh
FS4	Grapes, strawberries, blueberries	Grapes, ….<br>Berries, mixed<br>Blueberry, …<br>Mulberry, …<br>Strawberry, …<br>Raspberry, ….	Berries, mixed -strawberry, raspberry, blueberry, blackberry-, fresh, raw<br>Blueberry, fresh<br>Boysenberry, fresh<br>Grape, raw<br>Mulberry, fresh, raw<br>Raspberry<br>Strawberry
FS5	Melon eg. watermelon, rockmelon, honeydew melon	Melon, …	Melon

Table S2: Percentage Consumption of Selected Foods in Children aged 2-4 years and 5-8 years

		2 – 4 year old participants						5 – 8 year old participants					
ACAES FFQ Item		NNS (1995)			ANCNPAS (2007)			NNS (1995)			ANCNPAS (2007)		
		Female n=299	Male n=282	Persons n=581	Female n=715	Male n=776	Persons n=1491	Female n=282	Male n=342	Persons n=624	Female n=474	Male n=481	Persons n=955
**Drinks**													
D1	DIET soft drink eg. Diet coke	9 (3.0%)	11 (3.9%)	20 (3.4%)	18 (2.5%)	19 (2.4%)	37 (2.5%)	22 (7.8%)	29 (8.5%)	51 (8.2%)	21 (4.4%)	19 (4%)	40 (4.2%)
D2	Soft drink (NOT DIET) including flavoured mineral water	68 (22.7%)	83 (29.4%)	151 (26.0%)	76 (10.6%)	88 (11.3%)	164 (11.0%)	94 (33.3%)	136 (39.8%)	230 (36.9%)	98 (20.4%)	107 (22.2%)	205 (21.5%)
D3	Water - including bottled water. unflavoured mineral water, tap water	225 (75.3%)	193 (68.4%)	418 (71.9%)	681 (95.2%)	723 (93.2%)	1404 (94.2%)	269 (95.3%)	281 (82.2%)	550 (88.1%)	458 (95.2%)	455 (94.6%)	913 (95.6%)
D4	Fruit juice-based drinks	110 (36.8%)	103 (36.5%)	213 (36.7%)	337 (47.1%)	353 (45.5%)	690 (46.3%)	96 (34.0%)	111 (32.5%)	207 (33.2%)	211 (43.9%)	241 (50.1%)	452 (47.3%)
D5	Cordial or 'make up'	182 (60.9%)	148 (52.5%)	330 (56.8%)	126 (17.6%)	178 (22.9%)	304 (20.4%)	218 (77.3%)	190 (55.6%)	408 (65.4%)	102 (21.2%)	99 (20.6%)	201 (21.0%)
D6	Tea or Coffee	18 (6.0%)	18 (6.4%)	36 (6.2%)	21 (2.9%)	28 (3.6%)	49 (3.3%)	27 (9.6%)	22 (6.4%)	49 (7.9%)	29 (6%)	31 (6.4%)	60 (6.3%)
D7	Beer	2 (0.7%)	2 (0.7%)	4 (0.7%)	0	0	0	1 (0.4%)	0 (0%)	1 (0.2%)	0 (0%)	0 (0%)	0 (0%)
D8	Wine or wine coolers	0 (0%)	1 (0.4%)	1 (0.2%)	7 (1%)	8 (1%)	15 (1%)	0 (0%)	0 (0%)	0 (0%)	1 (0.2%)	2 (0.4%)	3 (0.3%)
D9	Spirits	0 (0%)	0 (0%)	0 (0%)	0 (0%)	0 (0%)	0 (0%)	0 (0%)	0 (0%)	0 (0%)	0 (0%)	0 (0%)	0 (0%)
**Dairy foods:**													
DF1 + DF3	Soy Milk	4 (1.3%)	14 (5.0%)	18 (3.1%)	26 (3.6%)	29 (3.7%)	55 (3.7%)	6 (2.1%)	5 (1.5%)	11 (1.8%)	9 (1.9%)	5 (1%)	14 (1.5%)

Table S2: Percentage Consumption of Selected Foods in Children aged 2-4 years and 5-8 years (cont)

ACAES FFQ Item		2 – 4 year old participants						5 – 8 year old participants					
		NNS (1995)			ANCNPAS (2007)			NNS (1995)			ANCNPAS (2007)		
		Female n=299	Male n=282	Persons n=581	Female n=715	Male n=776	Persons n=1491	Female n=282	Male n=342	Persons n=624	Female n=474	Male n=481	Persons n=955
DF1 + DF3	Whole Milk	230 (76.9%)	206 (73.0%)	436 (75.0%)	480 (67.1%)	549 (70.7%)	1029 (69.0%)	198 (70.2%)	240 (70.2%)	438 (70.2%)	236 (49.8%)	270 (56.1%)	506 (53.0%)
DF1 + DF3	Reduced Fat Milk	30 (10.0%)	23 (8.2%)	53 (9.1%)	123 (17.2%)	128 (16.5%)	251 (16.8%)	55 (19.5%)	41 (12.0%)	96 (15.4%)	95 (20%)	102 (21.2%)	197 (20.6%)
DF1 + DF3	Skim Milk	2 (0.7%)	6 (2.1%)	8 (1.4%)	23 (3.2%)	19 (2.4%)	42 (2.8%)	6 (2.1%)	6 (1.8%)	12 (1.9%)	23 (4.9%)	23 (4.8%)	46 (4.8%)
DF2	Flavoured milk	24 (8.0%)	15 (5.3%)	39 (6.7%)	55 (7.7%)	75 (9.7%)	130 (8.7%)	23 (8.2%)	42 (12.3%)	65 (10.4%)	69 (14.6%)	55 (11.4%)	124 (13.0%)
DF4	Cream or sour cream	14 (4.7%)	6 (2.1%)	20 (3.4%)	25 (3.5%)	24 (3.1%)	49 (3.3%)	13 (4.6%)	9 (2.6%)	22 (3.5%)	21 (4.4%)	25 (5.2%)	46 (4.8%)
DF5	Ice cream	55 (18.4%)	47 (16.7%)	102 (17.6%)	120 (16.8%)	107 (13.8%)	227 (15.2%)	65 (23.0%)	73 (21.3%)	138 (22.1%)	117 (24.7%)	101 (20.1%)	218 (22.8%)
DF6	Frozen yoghurt	1 (0.3%)	1 (0.4%)	2 (0.3%)	8 (1.1%)	3 (0.4%)	11 (0.7%)	3 (1.1%)	0 (0%)	3 (0.5%)	6 (1.3%)	5 (1%)	11 (1.2%)
DF7	Yoghurt (not frozen) plain or flavoured	33 (11.0%)	38 (13.5%)	71 (12.2%)	256 (35.8%)	272 (35.1%)	528 (35.4%)	28 (9.9%)	20 (5.8%)	48 (7.7%)	96 (20.3%)	105 (21.8%)	201 (21.0%)
DF8	Cottage cheese or ricotta	0 (0%)	0 (0%)	0 (0%)	4 (0.6%)	4 (0.5%)	8 (0.5%)	2 (0.7%)	1 (0.3%)	3 (0.5%)	0 (0%)	3 (0.6%)	3 (0.3%)
DF9	Cheese including cheese on sandwiches, biscuits or on toast	97 (32.4%)	99 (35.1%)	196 (33.7%)	343 (48%)	371 (47.8%)	714 (47.9%)	114 (40.4%)	112 (32.7%)	226 (36.2%)	177 (37.3%)	198 (41.2%)	375 (39.3%)
DF10	Cheese spread, cream cheese	28 (9.4%)	21 (7.4%)	49 (8.4%)	43 (6%)	42 (5.4%)	85 (5.7%)	23 (8.2%)	28 (8.2%)	51 (8.2%)	24 (5.1%)	15 (3.1%)	39 (4.1%)
**Breads and Cereal:**													

Table S2: Percentage Consumption of Selected Foods in Children aged 2-4 years and 5-8 years (cont)

		2 – 4 year old participants						5 – 8 year old participants					
ACAES FFQ Item		NNS (1995)			ANCNPAS (2007)			NNS (1995)			ANCNPAS (2007)		
		Female n=299	Male n=282	Persons n=581	Female n=715	Male n=776	Persons n=1491	Female n=282	Male n=342	Persons n=624	Female n=474	Male n=481	Persons n=955
B1	Muesli	4 (1.3%)	6 (2.1%)	10 (1.7%)	12 (1.7%)	13 (1.7%)	25 (1.7%)	7 (2.5%)	8 (2.3%)	15 (2.4%)	4 (0.8%)	3 (0.6%)	7 (0.7%)
B2	Cooked porridge	14 (4.7%)	9 (3.2%)	23 (4.0%)	55 (7.7%)	55 (7.1%)	110 (7.4%)	10 (3.5%)	13 (3.8%)	23 (3.7%)	29 (6.1%)	16 (3.3%)	45 (4.7%)
B3	Breakfast cereal	190 (63.5%)	177 (62.8%)	367 (63.2%)	418 (58.5%)	462 (59.5%)	880 (59%)	194 (68.8%)	208 (60.8%)	402 (64.4%)	240 (50.6%)	312 (64.9%)	552 (57.8%)
B5	Bread, pita bread, roll – White	204 (68.2%)	202 (71.6%)	406 (69.9%)	336 (47%)	395 (50.9%)	731 (49%)	243 (86.2%)	254 (74.3%)	497 (79.6%)	271 (57.2%)	273 (56.7%)	544 (57%)
B5	Bread, pita bread, roll – Mixed Grain	22 (7.4%)	21 (7.4%)	43 (7.4%)	84 (11.7%)	122 (15.7%)	206 (13.8%)	25 (8.9%)	23 (6.7%)	48 (7.7%)	51 (10.8%)	45 (9.4%)	96 (10.1%)
B5	Bread, pita bread, roll - Wholemeal	48 (16.1%)	42 (14.9%)	90 (15.5%)	198 (27.7%)	179 (23.1%)	377 (25.3%)	32 (11.3%)	53 (15.5%)	85 (13.6%)	85 (17.9%)	107 (22.2%)	192 (20.1%)
B6	English muffin, bagel or crumpet	6 (2.0%)	10 (3.5%)	16 (2.8%)	34 (4.8%)	32 (4.1%)	66 (4.4%)	9 (31.9%)	15 (4.4%)	24 (3.8%)	18 (3.8%)	17 (3.5%)	35 (3.7%)
B7	Rice	32 (10.7%)	29 (10.3%)	61 (10.5%)	112 (15.7%)	115 (14.8%)	227 (15.2%)	27 (9.6%)	40 (11.7%)	67 (10.7%)	68 (14.3%)	59 (12.3%)	127 (13.3%)
B8	Other grains eg. cous cous, burghul	0 (0%)	0 (0%)	0 (0%)	7 (1%)	5 (0.6%)	12 (0.8%)	1 (0.4%)	1 (0.3%)	2 (0.3%)	2 (0.4%)	1 (0.2%)	3 (0.3%)
B9	Noodles	17 (5.7%)	16 (5.7%)	33 (5.7%)	9 (1.3%)	19 (2.4%)	28 (1.9%)	17 (6.0%)	19 (5.6%)	36 (5.8%)	7 (1.5%)	9 (1.9%)	16 (1.7%)
B10	Pasta	35 (11.7%)	39 (13.8%)	74 (12.7%)	208 (29.1%)	232 (29.9%)	440 (29.5%)	43 (15.2%)	28 (8.2%)	71 (11.4%)	94 (19.8%)	122 (25.4%)	216 (22.6%)
**Sweets and Snacks:**													

Table S2: Percentage Consumption of Selected Foods in Children aged 2-4 years and 5-8 years (cont)

ACAES FFQ Item		2 – 4 year old participants						5 – 8 year old participants					
		NNS (1995)			ANCNPAS (2007)			NNS (1995)			ANCNPAS (2007)		
		Female n=299	Male n=282	Persons n=581	Female n=715	Male n=776	Persons n=1491	Female n=282	Male n=342	Persons n=624	Female n=474	Male n=481	Persons n=955
S1	Cakes, sweet muffins, scones, pikelets, pancakes, hot cakes	71 (23.7%)	51 (18.1%)	122 (21.0%)	170 (23.8%)	193 (24.9%)	363 (24.3%)	76 (27.0%)	96 (28.1%)	172 (27.6%)	145 (30.6%)	123 (25.6%)	268 (28.1%)
S2	Sweet pies or sweet pastries	9 (3.0%)	15 (5.3%)	24 (4.1%)	31 (4.3%)	38 (4.9%)	69 (4.6%)	28 (9.9%)	22 (6.4%)	50 (8.0%)	25 (5.3%)	28 (5.8%)	53 (5.5%)
S3	Other puddings or desserts (not ice cream)	51 (17.1%)	39 (13.8%)	90 (15.5%)	60 (8.4%)	78 (10.1%)	138 (9.3%)	34 (12.1%)	41 (12.0%)	75 (12.0%)	39 (8.2%)	37 (7.7%)	76 (8%)
S4	Plain sweet biscuits	67 (22.4%)	70 (24.8%)	137 (23.6%)	161 (22.5%)	155 (20%)	316 (21.2%)	71 (25.2%)	91 (26.6%)	162 (30.0%)	93 (19.6%)	88 (18.3%)	181 (19%)
S5	Cream or chocolate biscuits	44 (14.7%)	37 (13.1%)	81 (13.9%)	75 (10.5%)	95 (12.2%)	170 (11.4%)	68 (24.1%)	55 (16.1%)	123 (19.7%)	65 (13.7%)	71 (14.8%)	136 (14.2%)
S6	Dry or savoury biscuits, crispbread, crackers	76 (25.4%)	58 (20.6%)	134 (23.1%)	269 (37.6%)	268 (34.5%)	537 (36%)	82 (29.7%)	85 (24.9%)	167 (26.8%)	149 (31.4%)	139 (28.9%)	288 (30.2%)
S7	Savoury combination snacks	-	-	-	21 (2.9%)	22 (2.8%)	43 (2.9%)	-	-	-	25 (5.3%)	27 (5.6%)	52 (5.4%)
S8	Sweet combination snacks	-	-	-	-	-	-	-	-	-	-	-	-
S9	Snack noodles	1 (0.3%)	0 (0%)	1 (0.2%)	29 (4.1%)	25 (3.2%)	54 (3.6%)	1 (0.4%)	0 (0%)	1 (0.2%)	33 (7%)	31 (6.4%)	64 (6.7%)
S10	Fruit bars	26 (8.7%)	21 (7.4%)	47 (8.1%)	0 (0%)	0 (0%)	0 (0%)	32 (11.3%)	32 (9.4%)	64 (10.3%)	0 (0%)	0 (0%)	0 (0%)

Table S2: Percentage Consumption of Selected Foods in Children aged 2-4 years and 5-8 years (cont)

ACAES FFQ Item		2 – 4 year old participants						5 – 8 year old participants					
		NNS (1995)			ANCNPAS (2007)			NNS (1995)			ANCNPAS (2007)		
		Female n=299	Male n=282	Persons n=581	Female n=715	Male n=776	Persons n=1491	Female n=282	Male n=342	Persons n=624	Female n=474	Male n=481	Persons n=955
S11	Snack bars	-	-	-	42 (5.9%)	46 (5.9%)	88 (5.9%)	-	-	-	27 (5.7%)	39 (8.1%)	66 (6.9%)
S12	Muesli bars	20 (6.7%)	32 (11.3%)	52 (9.0%)	56 (7.8%)	59 (7.6%)	115 (7.7%)	44 (15.6%)	39 (11.4%)	83 (13.3%)	54 (11.4%)	63 (13.1%)	117 (12.3%)
**Main Meals:**													
M1	Mince dish	17 (5.7%)	23 (8.2%)	40 (6.9%)	121 (16.9%)	120 (15.5%)	241 (16.2%)	33 (11.7%)	36 (10.5%)	69 (11.1%)	53 (11.2%)	79 (16.4%)	132 (13.8%)
M2-3, 5 M11-12	Mixed meat dish	31 (10.3%)	30 (10.6%)	61 (10.5%)	12 (1.7%)	20 (2.5%)	32 (2.2%)	35 (12.4%)	35 (10.2%)	70 (11.2%)	6 (1.3%)	6 (1.2%)	12 (1.2%)
M4	Plain meat (beef or lamb) WITHOUT vegetables or salad	44 (14.7%)	46 (16.3%)	90 (15.5%)	116 (16.2%)	123 (15.9%)	239 (16%)	65 (23.0%)	56 (16.4%)	121 (19.4%)	65 (13.7%)	79 (16.4%)	144 (15.1%)
M6-7, M10	Mixed chicken dish	15 (5.0%)	15 (5.3%)	30 (5.2%)	29 (4.0%)	26 (3.4%)	55 (3.7%)	11 (3.9%)	8 (2.3%)	19 (3.0%)	17 (3.6%)	16 (3.3%)	33 (3.5%)
M8	Chicken crumbed	18 (6.0%)	13 (4.6%)	31 (5.3%)	69 (9.7%)	49 (6.3%)	118 (7.9%)	27 (9.6%)	35 (10.2%)	62 (9.9%)	57 (12%)	51 (10.6%)	108 (11.3%)
M9	Plain chicken WITHOUT vegetables	26 (8.7%)	26 (9.2%)	62 (10.7%)	115 (16.1%)	131 (16.9%)	246 (16.5%)	39 (13.8%)	53 (15.5%)	92 (14.7%)	78 (16.5%)	81 (16.8%)	159 (16.6%)
M13	Plain pork WITHOUT vegetables	8 (2.7%)	8 (2.8%)	16 (2.8%)	15 (2.1%)	21 (2.7%)	36 (2.4%)	8 (2.8%)	15 (4.4%)	23 (3.7%)	12 (2.5%)	14 (2.9%)	26 (2.7%)
M15	Liver – beef, calf, chicken	1 (0.3%)	2 (0.7%)	3 (0.5%)	0 (0%)	0 (0%)	0 (0%)	1 (0.4%)	1 (0.3%)	2 (0.3%)	2 (0.4%)	1 (0.2%)	3 (0.3%)

Table S2: Percentage Consumption of Selected Foods in Children aged 2-4 years and 5-8 years (cont)

ACAES FFQ Item		2 – 4 year old participants						5 – 8 year old participants					
		NNS (1995)			ANCNPAS (2007)			NNS (1995)			ANCNPAS (2007)		
		Female n=299	Male n=282	Persons n=581	Female n=715	Male n=776	Persons n=1491	Female n=282	Male n=342	Persons n=624	Female n=474	Male n=481	Persons n=955
M16-17	Fish	27 (9.0%)	13 (4.6%)	40 (6.9%)	61 (8.5%)	60 (7.7%)	121 (8.1%)	26 (9.2%)	16 (4.7%)	42 (6.7%)	41 (8.6%)	29 (6%)	70 (7.3%)
M18	Canned tuna, salmon, sardines including patties	4 (1.3%)	5 (1.8%)	9 (1.5%)	25 (3.5%)	29 (3.7%)	54 (3.6%)	13 (4.6%)	5 (1.5%)	18 (2.9%)	19 (4%)	17 (3.5%)	36 (3.8%)
M19	Other seafood	7 (2.3%)	1 (0.4%)	8 (1.4%)	9 (1.3%)	14 (1.8%)	23 (1.5%)	4 (1.4%)	12 (3.5%)	16 (2.6%)	10 (2.1%)	14 (2.9%)	24 (2.5%)
M20-21	Creamy or Clear soup	17 (5.7%)	19 (6.7%)	36 (6.2%)	49 (6.9%)	55 (7.1%)	104 (7%)	16 (5.7%)	31 (9.1%)	47 (7.5%)	34 (7.2%)	29 (6%)	63 (6.6%)
M22	Tacos, burritos, enchiladas	0 (0%)	1 (0.4%)	1 (0.2%)	7 (1%)	7 (0.9%)	14 (0.9%)	0 (0%)	0 (0%)	0 (0%)	10 (2.1%)	12 (2.5%)	22 (2.3%)
M23	Sausages, frankfurts, Pluto Pup	48 (16.1%)	41 (14.5%)	89 (15.3%)	89 (12.4%)	104 (13.4%)	193 (12.9%)	50 (17.7%)	51 (14.9%)	101 (16.2%)	59 (12.4%)	69 (14.3%)	128 (13.4%)
M24	Hamburger - all types	16 (5.4%)	21 (7.4%)	37 (6.4%)	7 (1%)	11 (1.4%)	18 (1.2%)	8 (2.8%)	25 (7.3%)	33 (5.3%)	15 (3.2%)	23 (4.8%)	38 (4.0%)
M25	Pizza	19 (6.4%)	13 (4.6%)	32 (5.5%)	27 (3.8%)	33 (4.3%)	60 (4.0%)	22 (7.8%)	39 (11.4%)	61 (9.8%)	34 (7.2%)	29 (6%)	63 (6.6%)
M26	Pie, sausage roll, chiko roll	34 (11.4%)	18 (6.4%)	52 (9.0%)	28 (3.9%)	53 (6.8%)	81 (5.4%)	37 (13.1%)	38 (11.1%)	75 (12.0%)	54 (11.4%)	42 (8.7%)	96 (10.1%)
M27	Hot dog	1 (0.3%)	1 (0.4%)	2 (0.3%)	-	-	-	0 (0%)	0 (0%)	0 (0%)	-	-	-
M28	Savoury pastries	6 (2.0%)	4 (1.4%)	10 (1.7%)	13 (1.8%)	24 (3.1%)	37 (2.5%)	6 (2.1%)	18 (5.3%)	24 (3.8%)	12 (2.5%)	13 (2.7%)	25 (2.6%)
M29	Hash browns, potato scallops	10 (3.3%)	7 (2.5%)	17 (2.9%)	7 (1%)	11 (1.4%)	18 (1.2%)	9 (3.2%)	8 (2.3%)	17 (2.7%)	9 (1.9%)	14 (2.9%)	23 (2.4%)
**Other Foods:**													

Table S2: Percentage Consumption of Selected Foods in Children aged 2-4 years and 5-8 years (cont)

ACAES FFQ Item		2 – 4 year old participants						5 – 8 year old participants					
		NNS (1995)			ANCNPAS (2007)			NNS (1995)			ANCNPAS (2007)		
		Female n=299	Male n=282	Persons n=581	Female n=715	Male n=776	Persons n=1491	Female n=282	Male n=342	Persons n=624	Female n=474	Male n=481	Persons n=955
O1	Chips (not potato)	31 (10.4%)	38 (13.5%)	69 (11.9%)	61 (8.5%)	75 (9.7%)	136 (9.1%)	53 (18.8%)	55 (16.1%)	108 (17.3%)	47 (9.9%)	65 (13.5%)	112 (11.7%)
O2	Potato chips or crisps	29 (9.7%)	57 (20.2%)	86 (14.8%)	69 (9.7%)	70 (9%)	139 (9.3%)	80 (28.4%)	72 (21.1%)	152 (24.4%)	85 (17.9%)	97 (20.2%)	182 (19.1%)
O3	Ice block – creamy	13 (4.3%)	23 (8.2%)	36 (6.2%)	21 (2.9%)	26 (3.4%)	47 (3.2%)	32 (11.3%)	39 (11.4%)	71 (11.4%)	23 (4.9%)	23 (4.8%)	46 (4.8%)
O4	Ice block – water	32 (10.7%)	29 (10.3%)	61 (10.5%)	28 (3.9%)	36 (4.6%)	64 (4.3%)	37 (13.1%)	45 (13.2%)	82 (13.1%)	40 (8.4%)	35 (7.3%)	75 (7.9%)
O5	Chocolate	78 (26.1%)	73 (25.9%)	151 (26.0%)	142 (19.9%)	145 (18.7%)	287 (19.2%)	99 (35.1%)	108 (31.6%)	207 (33.2%)	130 (27.4%)	128 (26.6%)	258 (27.0%)
O6	Lollies without chocolate	61 (20.4%)	56 (19.9%)	117 (20.1%)	114 (15.9%)	104 (13.4%)	218 (14.6%)	80 (28.4%)	77 (22.5%)	157 (25.2%)	111 (23.4%)	103 (21.4%)	214 (22.4%)
O7	Low fat salad dressing or mayonnaise	0 (0%)	2 (0.7%)	2 (0.3%)	11 (1.5%)	10 (1.3%)	21 (1.4%)	3 (1.1%)	9 (2.6%)	12 (1.9%)	5 (1.1%)	11 (2.3%)	16 (1.7%)
O8	Salad dressing or mayonnaise - not low fat	15 (5.0%)	11 (3.9%)	26 (4.5%)	34 (4.8%)	24 (3.1%)	58 (3.9%)	26 (9.2%)	18 (5.3%)	44 (7.1%)	21 (4.4%)	24 (5%)	45 (4.7%)
O9	Nuts	9 (3.0%)	7 (2.5%)	16 (2.8%)	34 (4.8%)	24 (3.1%)	58 (3.9%)	14 (5.0%)	13 (3.8%)	27 (4.3%)	18 (3.8%)	17 (3.5%)	35 (3.7%)
O10	Jam, honey, golden syrup, marmalade	44 (14.7%)	41 (14.5%)	85 (14.6%)	180 (25.2%)	211 (27.2%)	391 (26.2%)	45 (16.0%)	62 (18.1%)	107 (17.1%)	104 (21.9%)	102 (21.2%)	206 (21.6%)
O11	Peanut butter, Nutella	68 (22.7%)	72 (25.5%)	140 (24.1%)	97 (13.6%)	105 (13.5%)	202 (13.5%)	69 (24.5%)	81 (23.7%)	150 (24.0%)	76 (16%)	78 (16.2%)	154 (16.1%)
O12	Vegemite, Mighty Mite, Promite, Marmite	-	-	-	214 (30%)	278 (35.8%)	492 (33%)	-	-	-	124 (26.2%)	112 (23.3%)	236 (24.7%)

Table S2: Percentage Consumption of Selected Foods in Children aged 2-4 years and 5-8 years (cont)

ACAES FFQ Item		2 – 4 year old participants						5 – 8 year old participants					
		NNS (1995)			ANCNPAS (2007)			NNS (1995)			ANCNPAS (2007)		
		Female n=299	Male n=282	Persons n=581	Female n=715	Male n=776	Persons n=1491	Female n=282	Male n=342	Persons n=624	Female n=474	Male n=481	Persons n=955
O13	Tomato sauce, barbecue sauce	81 (27.1%)	65 (23.0%)	146 (25.1%)	134 (18.7%)	158 (20.4%)	292 (19.6%)	96 (34.0%)	113 (33.0%)	209 (33.5%)	126 (26.6%)	121 (25.2%)	247 (25.8%)
O14	Devon, salami	36 (12.0%)	28 (9.9%)	64 (11.0%)	48 (6.7%)	57 (7.3%)	105 (7%)	30 (10.6%)	29 (8.5%)	59 (9.5%)	55 (11.6%)	37 (7.7%)	92 (9.6%)
O15	Bacon, ham	40 (13.4%)	45 (16.0%)	85 (14.6%)	132 (18.5%)	148 (19.1%)	280 (18.8%)	49 (17.4%)	60 (17.5%)	109 (17.5%)	94 (19.8%)	112 (23.3%)	206 (21.5%)
O16	Eggs	37 (12.4%)	27 (9.6%)	64 (11.0%)	103 (14.4%)	100 (12.9%)	203 (13.6%)	33 (11.7%)	44 (12.9%)	77 (12.3%)	59 (12.4%)	65 (13.5%)	124 (13.0%)
O17	Jelly	6 (2.0%)	16 (5.7%)	22 (3.8%)	17 (2.4%)	30 (3.9%)	47 (3.2%)	10 (3.5%)	8 (2.3%)	18 (2.9%)	20 (4.2%)	11 (2.3%)	31 (3.2%)
**Fruit and Vegetables**													
F1-2	Hot chips bought from a shop or cooked at home	65 (21.7%)	67 (23.8%)	132 (22.7%)	112 (15.7%)	113 (14.6%)	225 (15.1%)	67 (23.8%)	87 (25.4%)	154 (24.7%)	101 (21.3%)	96 (20%)	197 (20.6%)
F3	Potato boiled, mashed, baked	90 (30.1%)	78 (27.7%)	168 (28.9%)	184 (25.7%)	203 (26.2%)	387 (30%)	98 (34.8%)	95 (27.8%)	193 (30.9%)	108 (22.8%)	102 (21.2%)	210 (22.0%)
F4	Pumpkin	20 (6.7%)	25 (8.9%)	45 (7.7%)	64 (9%)	59 (7.6%)	123 (8.2%)	26 (9.2%)	18 (5.3%)	44 (7.1%)	25 (5.3%)	27 (5.6%)	52 (5.4%)
F5	Sweet potato	1 (0.3%)	4 (1.4%)	5 (0.9%)	37 (5.2%)	30 (3.9%)	67 (4.5%)	1 (0.4%)	0 (0%)	1 (0.2%)	8 (1.7%)	12 (2.5%)	20 (2.1%)
F6	Cauliflower	10 (3.3%)	5 (1.8%)	15 (2.6%)	50 (7%)	41 (5.3%)	91 (6.1%)	15 (5.3%)	11 (3.2%)	26 (4.2%)	24 (5.1%)	27 (5.6%)	51 (5.3%)
F7	Green beans	28 (9.4%)	18 (6.4%)	46 (7.9%)	51 (7.1%)	59 (7.6%)	110 (7.3%)	29 (10.3%)	24 (7.0%)	53 (8.5%)	36 (7.6%)	27 (5.6%)	63 (6.6%)
F8	Spinach	4 (1.3%)	5 (1.8%)	9 (1.5%)	22 (3.1%)	13 (1.7%)	35 (2.3%)	6 (2.1%)	3 (0.9%)	9 (1.4%)	10 (2.1%)	9 (1.9%)	19 (2.0%)
F9	Cabbage or brussel sprouts	5 (1.7%)	10 (3.5%)	15 (2.6%)	15 (2.1%)	21 (2.7%)	36 (2.4%)	12 (4.3%)	13 (3.8%)	25 (4.0%)	12 (2.5%)	10 (2.1%)	22 (2.3%)

Table S2: Percentage Consumption of Selected Foods in Children aged 2-4 years and 5-8 years (cont)

ACAES FFQ Item		2 – 4 year old participants						5 – 8 year old participants					
		NNS (1995)			ANCNPAS (2007)			NNS (1995)			ANCNPAS (2007)		
		Female n=299	Male n=282	Persons n=581	Female n=715	Male n=776	Persons n=1491	Female n=282	Male n=342	Persons n=624	Female n=474	Male n=481	Persons n=955
F10	Peas	47 (15.7%)	49 (17.4%)	96 (16.5%)	107 (15%)	139 (17.9%)	246 (16.5%)	53 (18.8%)	43 (12.6%)	96 (15.4%)	58 (12.2%)	53 (11%)	111 (11.6%)
F11	Broccoli	24 (8.0%)	21 (7.4%)	45 (7.7%)	114 (15.(%)	105 (13.5%)	219 (14.7%)	26 (9.2%)	27 (7.9%)	53 (8.5%)	60 (12.7%)	66 (13.7%)	126 (13.2%)
F12	Carrots	88 (29.4%)	84 (29.8%)	172 (29.6%)	228 (31.9%)	268 (34.5%)	496 (33.3%)	103 (36.5%)	96 (28.1%)	199 (31.9%)	153 (32.3%)	132 (27.4%)	285 (29.8%)
F13	Zucchini, eggplant, squash	11 (3.7%)	7 (2.5%)	18 (3.1%)	38 (5.3%)	49 (6.3%)	87 (5.8%)	9 (3.2%)	8 (2.3%)	17 (2.7%)	16 (3.4%)	17 (3.5%)	33 (3.5%)
F14	Capsicum	8 (2.7%)	17 (6.0%)	25 (4.3%)	39 (5.5%)	49 (6.3%)	88 (5.9%)	21 (7.4%)	17 (5.0%)	38 (6.1%)	35 (7.4%)	22 (4.6%)	57 (6.0%)
F15	Corn, sweetcorn, corn on the cob	30 (10.0%)	14 (5.0%)	44 (7.6%)	104 (14.5%)	117 (15.1%)	221 (14.8%)	22 (7.8%)	28 (8.2%)	50 (8.0%)	57 (12%)	45 (9.4%)	102 (10.7%)
F16	Mushrooms	15 (5.0%)	17 (6.0%)	32 (5.5%)	38 (5.3%)	45 (5.8%)	83 (5.6%)	11 (3.9%)	13 (3.8%)	24 (3.8%)	17 (3.6%)	15 (3.1%)	32 (3.4%)
F17	Tomatoes	42 (14.0%)	31 (11.0%)	73 (12.6%)	111 (15.5%)	102 (13.1%)	213 (14.3%)	63 (22.3%)	39 (11.4%)	102 (16.3%)	64 (13.5%)	66 (13.7%)	130 (13.6%)
F18	Lettuce	35 (11.7%)	27 (9.6%)	62 (10.7%)	47 (6.6%)	31 (4%)	78 (5.2%)	61 (21.6%)	52 (15.2%)	113 (18.1%)	52 (11%)	65 (13.5%)	117 (12.3%)
F19	Celery, cucumber	23 (7.7%)	26 (9.2%)	49 (8.4%)	81 (11.3%)	74 (9.5%)	155 (10.4%)	38 (13.5%)	30 (8.8%)	68 (10.9%)	61 (12.9%)	57 (11.9%)	118 (12.4%)
F20	Avocado	1 (0.3%)	3 (1.1%)	4 (0.7%)	33 (4.6%)	24 (3.1%)	57 (3.8%)	1 (0.4%)	0 (0%)	1 (0.2%)	12 (2.5%)	10 (2.1%)	22 (2.3%)
F21	Onion, spring onion, leek	11 (3.7%)	9 (3.2%)	20 (3.4%)	98 (13.7%)	124 (16%)	222 (14.9%)	17 (6.0%)	33 (9.6%)	50 (8.0%)	61 (12.9%)	60 (12.5%)	121 (12.8%)
F22	Soybeans, tofu	0 (0%)	3 (1.1%)	3 (0.5%)	5 (0.7%)	5 (0.6%)	10 (0.7%)	0 ((0%)	0 (0%)	0 (0%)	1 (0.2%)	1 (0.2%)	2 (0.2%)
F23	Baked beans	9 (3.0%)	12 (4.3%)	21 (3.6%)	30 (4.2%)	30 (3.9%)	60 (4.0%)	7 (2.5%)	9 (2.6%)	16 (2.6%)	14 (3%)	14 (2.9%)	28 (2.9%)

Table S2: Percentage Consumption of Selected Foods in Children aged 2-4 years and 5-8 years (cont)

ACAES FFQ Item		2 – 4 year old participants: NNS (1995) Female n=299	Male n=282	Persons n=581	ANCNPAS (2007) Female n=715	Male n=776	Persons n=1491	5 – 8 year old participants: NNS (1995) Female n=282	Male n=342	Persons n=624	ANCNPAS (2007) Female n=474	Male n=481	Persons n=955
F24	Other beans, lentils	4 (1.3%)	8 (2.8%)	12 (2.1%)	18 (2.5%)	10 (1.3%)	28 (1.9%)	3 (1.1%)	3 (0.9%)	6 (1.0%)	7 (1.5%)	9 (1.9%)	16 (1.7%)
F25	Canned fruit	6 (2.0%)	2 (0.7%)	8 (1.4%)	49 (6.9%)	52 (6.7%)	101 (6.8%)	5 (1.8%)	2 (0.6%)	7 (1.1%)	25 (5.3%)	28 (5.8%)	53 (5.5%)
F26	Fruit salad	8 (2.7%)	7 (2.5%)	15 (2.6%)	22 (3.1%)	34 (4.4%)	56 (3.8%)	6 (2.1%)	7 (2.0%)	13 (2.1%)	9 (1.9%)	6 (1.2%)	15 (1.6%)
F27	Dried fruit	30 (10.0%)	24 (8.5%)	54 (9.3%)	99 (13.8%)	133 (17.1%)	232 (15.6%)	26 (9.2%)	7 (2.0%)	33 (5.3%)	28 (5.9%)	32 (6.7%)	60 (6.3%)
F28	Apple or pear	116 (38.8%)	129 (45.7%)	245 (42.2%)	347 (48.5%)	398 (51.3%)	745 (50%)	139 (49.3%)	108 (31.6%)	247 (39.6%)	184 (38.8%)	179 (37.2%)	363 (38.0%)
F29	Orange, mandarin, grapefruit	57 (19.1%)	50 (17.7%)	107 (18.4%)	159 (22.2%)	171 (22%)	330 (22.1%)	58 (20.6%)	55 (16.1%)	113 (18.1%)	95 (20%)	83 (17.3%)	178 (18.6%)
F30	Banana	78 (26.1%)	71 (25.2%)	149 (25.6%)	280 (39.2%)	253 (32.6%)	533 (35.7%)	66 (23.4%)	64 (18.7%)	130 (20.8%)	108 (22.8%)	118 (24.5%)	226 (23.7%)
**Seasonal Fruits:**													
FS1	Peach, nectarine, plum or apricot	24 (8.0%)	19 (6.7%)	43 (7.4%)	9 (1.3%)	10 (1.3%)	19 (1.3%)	25 (8.9%)	26 (7.6%)	51 (8.2%)	16 (3.4%)	10 (2.1%)	26 (2.7%)
FS2	Mango or paw-paw	1 (0.3%)	3 (1.1%)	4 (0.7%)	5 (0.7%)	2 (0.3%)	7 (0.5%)	3 (1.1%)	1 (0.3%)	4 (0.6%)	2 (0.4%)	0 (0%)	2 (0.2%)
FS3	Pineapple	2 (1.0%)	4 (1.4%)	6 (1.0%)	13 (1.8%)	6 (0.8%)	19 (1.3%)	9 (3.2%)	7 (2.0%)	16 (2.6%)	2 (0.4%)	5 (1%)	7 (0.7%)
FS4	Grapes, strawberries, blueberries	23 (7.7%)	18 (6.4%)	41 (7.1%)	153 (21.4%)	144 (18.6%)	297 (19.9%)	29 (10.3%)	21 (6.1%)	50 (8.0%)	61 (12.9%)	49 (10.2%)	110 (11.5%)
FS5	Melon	14 (4.7%)	9 (3.2%)	23 (4.0%)	64 (9%)	92 (11.9%)	156 (10.5%)	14 (5.0%)	12 (3.5%)	26 (4.2%)	45 (9.5%)	40 (8.3%)	85 (8.9%)

Table S3: Percentage Consumption of Selected Foods in Children aged 9-12 years and 13-16 years

ACAES FFQ Item		9 – 12 year old participants						13 – 16 year old participants					
		NNS (1995)			ANCNPAS (2007)			NNS (1995)			ANCNPAS (2007)		
		Female n=253	Male n=291	Persons n=544	Female n=509	Male n=457	Persons n=966	Female n=217	Male n=232	Persons n=449	Female n=694	Male n=695	Persons n=1389
**Drinks**													
D1	DIET soft drink	21 (8.3%)	26 (8.9%)	47 (8.6%)	28 (5.5%)	24 (5.3%)	52 (5.4%)	24 (11.1%)	17 (7.3%)	41 (9.1%)	53 (7.6%)	69 (9.9%)	122 (8.8%)
D2	Soft drink (NOT DIET) including flavoured mineral water	112 (44.3%)	121 (41.6%)	233 (42.8%)	145 (28.5%)	148 (32.4%)	293 (30.3%)	110 (50.7%)	144 (62.1%)	254 (56.6%)	217 (31.3%)	280 (40.3%)	497 (35.8%)
D3	Water - including bottled water. unflavoured mineral water, tap water	248 (98.0%)	275 (94.5%)	523 (96.1%)	488 (95.9%)	439 (96.1%)	927 (96%)	214 (98.6%)	230 (99.1%)	444 (98.9%)	630 (90.8%)	623 (89.6%)	1253 (90.2%)
D4	Fruit juice-based drinks	94 (37.2%)	85 (29.2%)	179 (32.9%)	230 (45.2%)	203 (44.4%)	433 (44.8%)	90 (41.5%)	70 (30.2%)	160 (35.6%)	317 (45.7%)	289 (41.6%)	606 (43.6%)
D5	Cordial or 'make up'	159 (62.8%)	174 (59.8%)	333 (61.2%)	105 (20.6%)	104 (22.8%)	209 (21.6%)	97 (44.7%)	119 (51.3%)	216 (48.1%)	124 (17.9%)	154 (22.2%)	278 (20%)
D6	Tea or Coffee	57 (22.5%)	35 (12.0%)	92 (16.9%)	41 (8.1%)	27 (5.9%)	68 (7%)	66 (30.4%)	62 (26.7%)	128 (28.5%)	133 (19.2%)	118 (17%)	251 (18.1%)
D7	Beer	0 (0%)	0 (0%)	0 (0%)	0 (0%)	0 (0%)	0 (0%)	5 (2.3%)	10 (4.3%)	15 (3.3%)	0 (0%)	1 (0.1%)	1 (0.1%)
D8	Wine or wine coolers	2 (0.8%)	2 (0.7%)	4 (0.7%)	4 (0.8%)	2 (0.4%)	6 (0.6%)	3 (1.4%)	0 (0%)	3 (0.7%)	7 (1%)	7 (1%)	14 (1%)
D9	Spirits	1 (0.4%)	0 (0%)	1 (0.2%)	1 (0.2%)	0 (0%)	1 (0.1%)	4 (1.8%)	2 (0.9%)	6 (1.3%)	7 (1%)	2 (0.3%)	9 (0.6%)
**Dairy foods:**													
DF1 + DF3	Soy Milk	5 (2.0%)	3 (1.0%)	8 (1.5%)	6 (1.2%)	7 (1.5%)	13 (1.3%)	1 (0.5%)	2 (0.9%)	3 (0.7%)	12 (1.7%)	7 (1%)	19 (1.4%)

Table S3: Percentage Consumption of Selected Foods in Children aged 9-12 years and 13-16 years (cont)

ACAES FFQ Item		9 – 12 year old participants: NNS (1995) Female n=253	Male n=291	Persons n=544	ANCNPAS (2007) Female n=509	Male n=457	Persons n=966	13 – 16 year old participants: NNS (1995) Female n=217	Male n=232	Persons n=449	ANCNPAS (2007) Female n=694	Male n=695	Persons n=1389
DF1 + DF3	Whole Milk	166 (65.6%)	187 (64.2%)	353 (64.9%)	209 (41.2%)	221 (48.4%)	430 (44.5%)	115 (53%)	147 (63.4%)	262 (58.4%)	241 (34.7%)	337 (48.5%)	578 (41.6%)
DF1 + DF3	Reduced Fat Milk	66 (26.1%)	75 (25.8%)	141 (25.9%)	120 (23.6%)	117 (48.4%)	237 (24.5%)	55 (25.3%)	59 (25.4%)	114 (25.4%)	133 (19.2%)	145 (20.9%)	278 (20.0%)
DF1 + DF3	Skim Milk	7 (2.8%)	9 (3.1%)	16 (2.9%)	32 (6.3%)	17 (3.7%)	49 (5.1%)	6 (2.8%)	9 (3.9%)	15 (3.3%)	36 (5.2%)	23 (3.3%)	59 (4.2%)
DF2	Flavoured milk	22 (8.7%)	33 (11.3%)	55 (10.1%)	83 (16.3%)	75 (16.4%)	158 (16.4%)	25 (11.5%)	28 (12.1%)	53 (11.8%)	126 (18.2%)	128 (18.4%)	254 (18.3%)
DF4	Cream or sour cream	21 (8.3%)	16 (5.5%)	37 (6.8%)	26 (5.1%)	28 (6.1%)	54 (5.6%)	19 (8.8%)	9 (3.9%)	28 (6.2%)	46 (6.6%)	32 (4.6%)	78 (5.6%)
DF5	Ice cream	71 (28.0%)	77 (26.5%)	148 (27.2%)	116 (22.8%)	97 (21.2%)	213 (22.0%)	39 (18%)	54 (23.3%)	93 (20.7%)	100 (14.4%)	127 (18.3%)	227 (16.3%)
DF6	Frozen yoghurt	3 (1.2%)	4 (1.4%)	7 (1.3%)	4 (0.8%)	5 (1.1%)	9 (0.9%)	0 (0%)	2 (0.9%)	2 (0.4%)	6 (0.9%)	4 (0.6%)	10 (0.7%)
DF7	Yoghurt (not frozen) plain or flavoured	24 (9.4%)	21 (7.2%)	45 (8.3%)	73 (14.3%)	65 (14.2%)	138 (14.3%)	22 (10.1%)	19 (8.2%)	41 (9.1%)	94 (13.5%)	74 (10.6%)	168 (12.1%)
DF8	Cottage cheese or ricotta	2 (0.8%)	1 (0.3%)	3 (0.6%)	2 (0.4%)	1 (0.2%)	3 (0.3%)	4 (1.8%)	0 (0%)	4 (0.9%)	3 (0.4%)	1 (0.1%)	4 (0.3%)
DF9	Cheese including cheese on sandwiches, biscuits or on toast	104 (40.9%)	92 (31.6%)	196 (36.0%)	208 (40.9%)	166 (36.3%)	374 (38.7%)	91 (41.9%)	99 (42.7%)	190 (42.3%)	256 (36.9%)	245 35.3%)	501 (36.1%)
DF10	Cheese spread, cream cheese	21 (8.3%)	14 (4.8%)	35 (6.4%)	15 (2.9%)	10 (2.2%)	25 (2.6%)	22 (10.1%)	10 (4.3%)	32 (7.1%)	16 (2.3%)	20 (2.9%)	36 (2.6%)
**Breads and Cereal:**													
B1	Muesli	9 (3.5%)	11 (3.8%)	20 (3.7%)	11 (2.2%)	3 (0.7%)	14 (1.4%)	10 (4.6%)	1 (0.4%)	11 (2.4%)	8 (1.2%)	9 (1.3%)	17 (1.2%)

Table S3: Percentage Consumption of Selected Foods in Children aged 9-12 years and 13-16 years (cont)

ACAES FFQ Item		9 – 12 year old participants						13 – 16 year old participants					
		NNS (1995)			ANCNPAS (2007)			NNS (1995)			ANCNPAS (2007)		
		Female n=253	Male n=291	Persons n=544	Female n=509	Male n=457	Persons n=966	Female n=217	Male n=232	Persons n=449	Female n=694	Male n=695	Persons n=1389
B2	Cooked porridge	5 (2.0%)	4 (1.4%)	9 (1.7%)	23 (4.5%)	26 (5.7%)	49 (5.1%)	6 (2.8%)	6 (2.6%)	12 (2.7%)	30 (4.3%)	20 (2.9%)	50 (3.6%)
B3	Breakfast cereal	143 (56.3%)	203 (69.8%)	346 (63.6%)	191 (37.5%)	247 (54%)	438 (45.3%)	97 (44.7%)	157 (67.7%)	254 (56.6%)	205 (29.5%)	357 (51.4%)	562 (40.5%)
B5	Bread, pita bread, roll – White	238 (93.7%)	238 (81.8%)	476 (87.5%)	284 (55.8%)	266 (58.2%)	550 (57%)	154 (71%)	187 (80.6%)	341 (75.9%)	342 (49.3%)	391 (56.3%)	733 (52.8%)
B5	Bread, pita bread, roll – Mixed Grain	21 (8.3%)	17 (5.8%)	38 (7.0%)	51 (10%)	32 (7%)	83 (8.6%)	29 (13.4%)	17 (7.3%)	46 (10.2%)	80 (11.5%)	61 (8.8%)	141 (10.2%)
B5	Bread, pita bread, roll - Wholemeal	37 (14.6%)	46 (15.8%)	83 (15.3%)	73 (14.3%)	72 (15.8%)	145 (15.0%)	31 (14.3%)	21 (9.1%)	52 (11.6%)	106 (15.3%)	91 (13.1%)	197 (14.2%)
B6	English muffin, bagel or crumpet	10 (3.9%)	12 (4.1%)	22 (4.0%)	25 (4.9%)	21 (4.6%)	46 (4.8%)	11 (5.1%)	9 (3.9%)	20 (4.5%)	35 (5.0%)	42 (6.0%)	77 (5.5%)
B7	Rice	34 (13.4%)	30 (10.3%)	64 (11.8%)	80 (15.7%)	65 (14.2%)	145 (15%)	22 (10.1%)	23 (9.9%)	45 (10.0%)	108 (15.6%)	121 (17.4%)	229 (16.5%)
B8	Other grains eg. cous cous, burghul	-	-	-	3 (0.6%)	2 (0.4%)	5 (0.5%)	-	-	-	4 (0.6%)	3 (0.4%)	7 (0.5%)
B9	Noodles	18 (7.1%)	14 (4.8%)	32 (5.9%)	11 (2.2%)	9 (2%)	20 (2.1%)	15 (6.9%)	12 (5.2%)	27 (6.0%)	14 (2.0%)	11 (1.6%)	25 (1.8%)
B10	Pasta	23 (9.1%)	29 (10.0%)	52 (9.6%)	106 (20.8%)	98 (21.4%)	204 (21.1%)	31 (14.3%)	22 (9.5%)	53 (11.8%)	142 (20.5%)	151 (21.7%)	293 (21.1%)
**Sweets and Snacks:**													
S1	Cakes, sweet muffins, scones, pikelets, pancakes, hot cakes	84 (33.1%)	87 (29.9%)	171 (31.4%)	145 (28.5%)	113 (24.7%)	258 (26.7%)	54 (24.9%)	45 (19.4%)	99 (22.0%)	180 (25.9%)	167 (24%)	347 (25%)
S2	Sweet pies or sweet pastries	32 (12.6%)	19 (6.5%)	51 (9.4%)	46 (9%)	39 (8.5%)	85 (8.8%)	27 (12.4%)	31 (13.4%)	58 (12.9%)	42 (6.1%)	51 (7.3%)	93 (6.7%)

Table S3: Percentage Consumption of Selected Foods in Children aged 9-12 years and 13-16 years (cont)

ACAES FFQ Item		9 – 12 year old participants NNS (1995) Female n=253	Male n=291	Persons n=544	ANCNPAS (2007) Female n=509	Male n=457	Persons n=966	13 – 16 year old participants NNS (1995) Female n=217	Male n=232	Persons n=449	ANCNPAS (2007) Female n=694	Male n=695	Persons n=1389
S3	Other puddings or desserts (not ice cream)	27 (10.6%)	31 (10.6%)	58 (10.7%)	43 (8.4%)	32 (7%)	75 (7.8%)	13 (6.0%)	18 (7.8%)	31 (6.9%)	43 (6.2%)	35 (5%)	78 (5.6%)
S4	Plain sweet biscuits	53 (20.9%)	57 (19.6%)	110 (20.2%)	81 (15.9%)	69 (15.1%)	150 (15.5%)	28 (12.9%)	36 (15.5%)	64 (14.3%)	92 (13.3%)	92 (13.2%)	184 (13.2%)
S5	Cream or chocolate biscuits	46 (18.1%)	60 (20.6%)	106 (19.5%)	64 (12.6%)	75 (16.4%)	139 (14.4%)	33 (15.2%)	40 (17.2%)	73 (16.3%)	78 (11.2%)	100 (14.4%)	178 (12.8%)
S6	Dry or savoury biscuits, crispbread, crackers	61 (24.0%)	68 (23.4%)	129 (23.7%)	134 (26.3%)	106 (23.2%)	240 (24.8%)	49 (22.6%)	38 (16.4%)	87 (19.4%)	124 (17.9%)	109 (15.7%)	233 (16.8%)
S7	Savoury combination snacks	-	-	-	15 (2.9%)	12 (2.6%)	27 (2.8%)	-	-	-	11 (1.6%)	13 (1.9%)	24 (1.7%)
S8	Sweet combination snacks	-	-	-	-	-	-	-	-	-	-	-	-
S9	Snack noodles	-	-	-	33 (6.5%)	38 (8.3%)	71 (7.3%)	-	-	-	52 (7.5%)	48 (6.9%)	100 (7.2%)
S10	Fruit bars	29 (11.4%)	24 (8.2%)	53 (9.7%)	0 (0%)	0 (0%)	0 (0%)	12 (5.5%)	12 (5.2%)	24 (5.3%)	0 (0%)	0 (0%)	0 (0%)
S11	Snack bars	-	-	-	19 (3.7%)	18 (3.9%)	37 (3.8%)	-	-	-	26 (3.7%)	32 (4.6%)	58 (4.2%)
S12	Muesli bars	26 (10.2%)	31 (10.7%)	57 (10.5%)	63 (12.4%)	60 (13.1%)	123 (12.7%)	13 (6.0%)	25 (10.8%)	38 (8.5%)	97 (14.0%)	114 (16.4%)	211 (15.2%)
**Main Meals:**													
M1	Mince dish	34 (13.4%)	36 (12.4%)	70 (12.9%)	63 (12.4%)	69 (15.1%)	132 (13.7%)	37 (17.1%)	40 (17.2%)	77 (17.1%)	84 (12.1%)	99 (14.2%)	183 (13.2%)

Table S3: Percentage Consumption of Selected Foods in Children aged 9-12 years and 13-16 years (cont)

ACAES FFQ Item		9 – 12 year old participants						13 – 16 year old participants					
		NNS (1995)			ANCNPAS (2007)			NNS (1995)			ANCNPAS (2007)		
		Female n=253	Male n=291	Persons n=544	Female n=509	Male n=457	Persons n=966	Female n=217	Male n=232	Persons n=449	Female n=694	Male n=695	Persons n=1389
M2-3, M5, M11-12	Mixed meat dish	32 (12.6%)	32 (11%)	64 (11.8%)	12 (2.4%)	8 (1.8%)	20 (2.0%)	26 (12.0%)	32 (13.8%)	58 (12.9%)	22 (3.2%)	18 (2.6%)	40 (2.9%)
M4	Plain meat (beef or lamb) WITHOUT vegetables or salad	76 (29.9%)	79 (27.1%)	155 (28.5%)	84 (16.5%)	91 (19.9%)	175 (18.1%)	67 (30.9%)	56 (24.1%)	123 (27.4%)	129 (18.6%)	148 (21.3%)	277 (19.9%)
M6-7, M10	Mixed chicken dish	9 (3.5%)	8 (2.7%)	17 (3.1%)	30 (5.9%)	20 (4.4%)	50 (5.2%)	14 (6.5%)	9 (3.9%)	23 (5.1%)	39 (5.6%)	58 (8.3%)	97 (6.9%)
M8	Chicken crumbed	15 (5.9%)	28 (9.6%)	43 (7.9%)	56 (11%)	41 (9%)	97 (10%)	22 (10.1%)	27 (11.6%)	49 (10.9%)	59 (8.5%)	69 (9.9%)	128 (9.2%)
M9	Plain chicken WITHOUT vegetables	53 (20.9%)	31 (10.7%)	84 (15.4%)	85 (16.7%)	89 (19.5%)	174 (18%)	43 (19.8%)	43 (18.5%)	86 (19.2%)	119 (17.1%)	137 (19.7%)	256 (18.4%)
M13	Plain pork WITHOUT vegetables	22 (8.7%)	16 (5.5%)	38 (7.0%)	16 (3.1%)	18 (3.9%)	34 (3.5%)	10 (4.6%)	5 (2.2%)	15 (3.3%)	29 (4.2%)	21 (3%)	50 (3.6%)
M15	Liver – beef, calf, chicken	0 (0%)	2 (0.7%)	2 (3.7%)	3 (0.6%)	0 (0%)	3 (0.3%)	1 (0.5%)	0 (0%)	1 (0.2%)	1 (0.1%)	1 (0.1%)	2 (0.1%)
M16-17	Fish	12 (4.7%)	17 (5.8%)	29 (5.3%)	28 (5.5%)	27 (5.9%)	55 (5.7%)	10 (4.6%)	22 (9.5%)	32 (7.1%)	31 (4.5%)	13 (1.9%)	44 (3.2%)
M18	Canned tuna, salmon, sardines including patties	8 (3.1%)	5 (1.7%)	13 (2.4%)	25 (4.9%)	16 (3.5%)	41 (4.2%)	6 (2.8%)	0 (0%)	6 (1.3%)	26 (3.7%)	20 (2.9%)	46 (3.3%)
M19	Other seafood	4 (1.6%)	6 (2.1%)	10 (1.8%)	9 (1.8%)	9 (2%)	18 (1.9%)	5 (2.3%)	4 (1.7%)	9 (2.0%)	12 (1.7%)	9 (1.3%)	21 (1.5%)
M20-21	Creamy or Clear soup	23 (9.1%)	27 (9.3%)	50 (9.2%)	44 (8.6%)	29 (6.3%)	73 (7.6%)	14 (6.5%)	11 (4.7%)	25 (5.6%)	71 (10.2%)	55 (7.9%)	126 (9.1%)
M22	Tacos, burritos, enchiladas	0 (0%)	0 (0%)	0 (0%)	15 (2.9%)	13 (2.8%)	28 (2.9%)	0 (0%)	1 (0.4%)	1 (0.2%)	8 (1.2%)	8 (1.2%)	16 (1.2%)

Table S3: Percentage Consumption of Selected Foods in Children aged 9-12 years and 13-16 years (cont)

ACAES FFQ Item		9 – 12 year old participants NNS (1995) Female n=253	Male n=291	Persons n=544	ANCNPAS (2007) Female n=509	Male n=457	Persons n=966	13 – 16 year old participants NNS (1995) Female n=217	Male n=232	Persons n=449	ANCNPAS (2007) Female n=694	Male n=695	Persons n=1389
M23	Sausages, frankfurts, Pluto Pup	46 (18.1%)	65 (22.3%)	111 (20.4%)	62 (12.2%)	68 (14.9%)	130 (13.5%)	22 (10.1%)	37 (15.9%)	59 (13.1%)	59 (8.5%)	93 (13.4%)	152 (10.9%)
M24	Hamburger - all types	22 (8.7%)	18 (6.2%)	40 (7.4%)	21 (4.1%)	31 (6.8%)	52 (5.4%)	24 (11.1%)	24 (10.3%)	48 (10.7%)	45 (6.5%)	41 (5.9%)	86 (6.2%)
M25	Pizza	27 (10.6%)	38 (13.1%)	65 (11.9%)	40 (7.9%)	35 (7.7%)	75 (7.8%)	21 (9.7%)	28 (12.1%)	49 (10.9%)	54 (7.8%)	73 (10.5%)	127 (9.1%)
M26	Pie, sausage roll, chiko roll	44 (17.3%)	40 (13.7%)	84 (15.4%)	49 (9.6%)	57 (12.5%)	106 (11.0%)	33 (15.2%)	47 (20.3%)	80 (17.8%)	68 (9.8%)	89 (12.8%)	157 (11.3%)
M27	Hot dog	-	-	-	-	-	-	-	-	-	-	-	-
M28	Savoury pastries	11 (4.3%)	10 (3.4%)	21 (3.9%)	28 (5.5%)	16 (3.5%)	44 (4.6%)	14 (6.5%)	17 (7.3%)	31 (6.9%)	35 (5.0%)	18 (2.6%)	53 (3.8%)
M29	Hash browns, potato scallops	11 (4.3%)	6 (2.1%)	17 (3.1%)	16 (3.1%)	7 (1.5%)	23 (2.4%)	8 (3.7%)	9 (3.9%)	17 (3.8%)	11 (1.6%)	19 (2.7%)	30 (2.2%)
**Other Foods:**													
O1	Chips (not potato)	59 (23.2%)	41 (14.1%)	100 (18.4%)	59 (11.6%)	54 (11.8%)	113 (11.7%)	35 (16.1%)	29 (12.5%)	64 (14.3%)	66 (9.5%)	52 (7.5%)	118 (8.5%)
O2	Potato chips or crisps	65 (25.6%)	57 (19.6%)	122 (22.4%)	95 (18.7%)	98 (21.4%)	193 (20%)	40 (18.4%)	47 (20.3%)	87 (19.4%)	125 (18%)	134 (19.2%)	259 (18.6%)
O3	Ice block – creamy	34 (13.4%)	32 (11.0%)	66 (12.1%)	28 (5.5%)	29 (6.3%)	57 (5.9%)	23 (10.6%)	20 (8.6%)	43 (9.6%)	36 (5.2%)	31 (4.5%)	67 (4.8%)
O4	Ice block – water	32 (12.6%)	28 (9.6%)	60 (11.0%)	29 (5.7%)	29 (6.3%)	58 (6%)	15 (6.9%)	11 (4.7%)	26 (5.8%)	25 (3.6%)	24 (3.5%)	49 (3.5%)
O5	Chocolate	92 (36.2%)	93 (32.0%)	185 (34.0%)	148 (29.1%)	129 (28.2%)	277 (28.7%)	83 (38.2%)	61 (26.3%)	144 (32.1%)	186 (26.8%)	145 (20.9%)	331 (23.8%)
O6	Lollies without chocolate	65 (25.6%)	58 (19.9%)	123 (22.6%)	107 (21%)	102 (22.3%)	209 (21.6%)	39 (18.0%)	28 (12.1%)	67 (14.9%)	141 (20.3%)	121 (17.4%)	262 (18.9%)

Table S3: Percentage Consumption of Selected Foods in Children aged 9-12 years and 13-16 years (cont)

ACAES FFQ Item		9 – 12 year old participants NNS (1995) Female n=253	Male n=291	Persons n=544	ANCNPAS (2007) Female n=509	Male n=457	Persons n=966	13 – 16 year old participants NNS (1995) Female n=217	Male n=232	Persons n=449	ANCNPAS (2007) Female n=694	Male n=695	Persons n=1389
O7	Low fat salad dressing or mayonnaise	8 (3.1%)	10 (3.4%)	18 (3.3%)	13 (2.6%)	5 (1.1%)	18 (1.9%)	6 (2.8%)	10 (4.3%)	16 (3.6%)	20 (2.9%)	28 (4%)	48 (3.5%)
O8	Salad dressing or mayonnaise - not low fat	29 (11.4%)	28 (9.6%)	57 (10.5%)	39 (7.7%)	39 (8.5%)	78 (8.1%)	28 (12.9%)	31 (13.4%)	59 (13.1%)	68 (9.8%)	74 (10.6%)	142 (10.2%)
O9	Nuts	11 (4.3%)	9 (3.1%)	20 (3.7%)	24 (4.7%)	16 (3.5%)	40 (4.1%)	11 (5.1%)	4 (1.7%)	15 (3.3%)	36 (5.2%)	25 (3.6%)	61 (4.4%)
O10	Jam, honey, golden syrup, marmalade	40 (15.7%)	66 (22.7%)	106 (19.5%)	90 (17.7%)	73 (16%)	163 (16.9%)	31 (14.3%)	21 (9.1%)	52 (11.6%)	123 (17.7%)	111 (16%)	234 (16.8%)
O11	Peanut butter, Nutella	56 (22.0%)	52 (17.9%)	108 (19.9%)	67 (13.2%)	76 (16.6%)	143 (14.8%)	13 (6.0%)	25 (10.8%)	38 (8.5%)	80 (11.5%)	102 (14.7%)	182 (13.1%)
O12	Vegemite, Mighty Mite, Promite, Marmite	-	-	-	111 (21.8%)	76 (16.6%)	187 (19.4%)	-	-	-	141 (20.3%)	97 (14%)	238 (17.1%)
O13	Tomato sauce, barbecue sauce	91 (35.8%)	112 (38.5%)	203 (37.3%)	131 (25.7%)	139 (30.4%)	270 (30%)	74 (34.1%)	98 (42.2%)	172 (38.3%)	159 (22.9%)	204 (29.4%)	363 (26.1%)
O14	Devon, salami	17 (6.7%)	42 (14.4%)	59 (10.8%)	30 (5.9%)	33 (7.2%)	63 (6.5%)	21 (9.7%)	22 (9.5%)	43 (9.6%)	37 (5.3%)	50 (7.2%)	87 (6.2%)
O15	Bacon, ham	56 (22.0%)	55 (18.9%)	111 (20.4%)	93 (18.3%)	90 (19.7%)	183 (18.9%)	43 (19.8%)	62 (26.7%)	105 (23.4%)	145 (20.9%)	156 (22.4%)	301 (21.7%)
O16	Eggs	40 (15.7%)	43 (14.8%)	83 (15.3%)	58 (11.4%)	60 (13.1%)	118 (12.2%)	21 (9.7%)	42 (18.1%)	63 (14.0%)	101 (14.6%)	86 (12.4%)	187 (13.5%)
O17	Jelly	9 (3.5%)	5 (1.7%)	14 (2.6%)	23 (4.5%)	9 (2%)	32 (3.3%)	5 (2.3%)	2 (0.9%)	7 (1.6%)	10 (1.4%)	6 (0.9%)	16 (1.2%)
**Fruit and Vegetables**													
F1-2	Hot chips bought from a shop or cooked at home	74 (29.1%)	86 (30.0%)	160 (29.4%)	104 (20.4%)	94 (20.6%)	198 (20.5%)	56 (25.8%)	78 (33.6%)	134 (29.8%)	167 (24.1%)	154 (22.2%)	321 (23.1%)

Table S3: Percentage Consumption of Selected Foods in Children aged 9-12 years and 13-16 years (cont)

ACAES FFQ Item		9 – 12 year old participants						13 – 16 year old participants					
		NNS (1995)			ANCNPAS (2007)			NNS (1995)			ANCNPAS (2007)		
		Female n=253	Male n=291	Persons n=544	Female n=509	Male n=457	Persons n=966	Female n=217	Male n=232	Persons n=449	Female n=694	Male n=695	Persons n=1389
F3	Potato boiled, mashed, baked	112 (44.1%)	115 (39.5%)	227 (41.7%)	125 (24.6%)	115 (25.2%)	240 (24.8%)	95 (43.8%)	77 (33.2%)	172 (38.3%)	158 (22.8%)	158 (22.7%)	316 (22.8%)
F4	Pumpkin	33 (13.0%)	37 (12.7%)	70 (12.9%)	38 (7.5%)	25 (5.5%)	63 (6.5%)	30 (13.8%)	23 (9.9%)	53 (11.8%)	50 (7.2%)	27 (3.9%)	77 (5.5%)
F5	Sweet potato	5 (2.0%)	1 (0.3%)	6 (1.1%)	11 (2.2%)	16 (3.5%)	27 (2.8%)	1 (0.5%)	5 (2.2%)	6 (1.3%)	22 (3.2%)	20 (2.9%)	42 (3%)
F6	Cauliflower	17 (6.7%)	20 (6.9%)	37 (6.8%)	28 (5.5%)	27 (5.9%)	55 (5.7%)	13 (6.0%)	13 (5.6%)	26 (2.8%)	37 (5.3%)	30 (4.3%)	67 (4.8%)
F7	Green beans	29 (11.4%)	35 (12.0%)	64 (11.8%)	51 (10%)	32 (7%)	83 (8.6%)	25 (11.5%)	21 (9.1%)	46 (10.2%)	61 (8.8%)	59 (8.5%)	120 (8.6%)
F8	Spinach	6 (2.4%)	6 (2.1%)	12 (2.2%)	13 (2.6%)	8 (1.8%)	21 (2.2%)	7 (3.2%)	2 (0.9%)	9 (2.0%)	21 (3.0%)	25 (3.6%)	46 (3.3%)
F9	Cabbage or brussel sprouts	19 (7.5%)	17 (5.8%)	36 (6.6%)	12 (2.4%)	15 (3.3%)	27 (2.8%)	14 (6.5%)	10 (4.3%)	24 (5.3%)	23 (3.3%)	26 (3.7%)	49 (3.5%)
F10	Peas	65 (25.6%)	54 (18.6%)	119 (21.9%)	69 (13.6%)	56 (12.3%)	125 (12.9%)	37 (17.1%)	40 (17.2%)	77 (17.1%)	80 (11.5%)	78 (11.2%)	158 (11.4%)
F11	Broccoli	27 (10.6%)	35 (12.0%)	62 (11.4%)	72 (14.1%)	57 (12.5%)	129 (13.4%)	27 (12.4%)	20 (8.6%)	47 (10.5%)	87 (12.5%)	81 (11.7%)	168 (12.1%)
F12	Carrots	98 (38.6%)	93 (32.0%)	191 (35.1%)	170 (33.4%)	136 (30%)	306 (31.7%)	87 (40.1%)	65 (28.0%)	152 (33.9%)	203 (29.3%)	181 (26%)	384 (27.6%)
F13	Zucchini, eggplant, squash	14 (5.5%)	11 (3.8%)	25 (4.6%)	21 (4.1%)	16 (3.5%)	37 (3.8%)	11 (5.1%)	10 (4.3%)	21 (4.7%)	24 (3.5%)	18 (2.6%)	42 (3%)
F14	Capsicum	24 (9.4%)	19 (6.5%)	43 (7.9%)	41 (8.1%)	35 (7.7%)	76 (7.9%)	28 (12.9%)	19 (8.2%)	47 (10.5%)	55 (7.9%)	49 (7.1%)	104 (7.5%)
F15	Corn, sweetcorn, corn on the cob	26 (10.2%)	20 (6.9%)	46 (8.5%)	53 (10.4%)	51 (11.2%)	104 (10.8%)	23 (10.6%)	10 (4.3%)	33 (7.3%)	58 (8.4%)	54 (7.8%)	112 (8.1%)
F16	Mushrooms	11 (4.3%)	9 (3.1%)	20 (3.7%)	22 (4.3%)	16 (3.5%)	38 (3.9%)	13 (6.0%)	14 (6.0%)	27 (6.0%)	41 (5.9%)	25 (3.6%)	66 (4.8%)

Table S3: Percentage Consumption of Selected Foods in Children aged 9-12 years and 13-16 years (cont)

ACAES FFQ Item		9 – 12 year old participants: NNS (1995) Female n=253	Male n=291	Persons n=544	ANCNPAS (2007) Female n=509	Male n=457	Persons n=966	13 – 16 year old participants: NNS (1995) Female n=217	Male n=232	Persons n=449	ANCNPAS (2007) Female n=694	Male n=695	Persons n=1389
F17	Tomatoes	68 (26.8%)	72 (24.7%)	140 (25.7%)	111 (21.8%)	79 (17.3%)	190 (19.7%)	73 (33.6%)	49 (21.1%)	122 (27.2%)	135 (19.5%)	142 (20.4%)	277 (19.9%)
F18	Lettuce	84 (33.1%)	66 (22.7%)	150 (27.6%)	92 (18.1%)	67 (14.7%)	159 (16.5%)	64 (29.5%)	60 (25.9%)	124 (27.6%)	122 (17.6%)	134 (19.3%)	256 (18.4%)
F19	Celery, cucumber	42 (16.5%)	41 (14.1%)	83 (15.3%)	68 (13.4%)	47 (10.3%)	115 (11.9%)	33 (15.2%)	17 (7.3%)	50 (11.1%)	94 (13.5%)	76 (10.9%)	170 (12.2%)
F20	Avocado	3 (1.2%)	1 (0.3%)	4 (0.7%)	17 (3.3%)	12 (2.6%)	29 (3.0%)	10 (4.6%)	2 (0.9%)	12 (2.7%)	33 (4.8%)	19 (2.7%)	52 (3.7%)
F21	Onion, spring onion, leek	29 (11.4%)	36 (12.4%)	65 (11.9%)	78 (15.3%)	72 (15.8%)	150 (15.5%)	30 (13.8%)	41 (17.7%)	71 (15.8%)	138 (19.9%)	165 (23.7%)	303 (21.8%)
F22	Soybeans, tofu	0 (0%)	0 (0%)	0 (0%)	2 (0.4%)	3 (0.7%)	5 (0.5%)	0 (0%)	0 (0%)	0 (0%)	5 (0.7%)	7 (1%)	12 (0.9%)
F23	Baked beans	9 (3.5%)	10 (3.4%)	19 (3.5%)	6 (1.2%)	10 (2.2%)	16 (1.7%)	6 (2.8%)	6 (2.6%)	12 (2.7%)	12 (1.7%)	12 (1.7%)	24 (1.7%)
F24	Other beans, lentils	2 (0.8%)	11 (3.8%)	13 (2.4%)	7 (1.4%)	11 (2.4%)	18 (1.9%)	3 (1.4%)	7 (3.0%)	10 (2.2%)	19 (2.7%)	12 (1.7%)	31 (2.2%)
F25	Canned fruit	2 (0.8%)	0 (0%)	2 (0.4%)	24 (4.7%)	18 (3.9%)	42 (4.3%)	1 (0.5%)	3 (1.3%)	4 (0.9%)	22 (3.2%)	19 (2.7%)	41 (3%)
F26	Fruit salad	6 (2.4%)	1 (0.3%)	7 (1.3%)	9 (1.8%)	8 (1.8%)	17 (1.8%)	2 (0.9%)	3 (1.3%)	5 (1.1%)	11 (1.6%)	11 (1.6%)	22 (1.6%)
F27	Dried fruit	8 (3.1%)	10 (3.4%)	18 (3.3%)	19 (3.7%)	18 (3.9%)	37 (3.8%)	7 (3.2%)	8 (3.4%)	15 (3.3%)	36 (5.2%)	28 (4%)	64 (4.6%)
F28	Apple or pear	118 (46.5%)	106 (36.4%)	224 (41.2%)	198 (38.9%)	152 (33.3%)	350 (36.2%)	73 (33.6%)	61 (26.3%)	134 (29.8%)	198 (28.5%)	173 (24.9%)	371 (26.7%)
F29	Orange, mandarin, grapefruit	43 (16.9%)	54 (18.6%)	97 (17.8%)	84 (16.5%)	66 (14.4%)	150 (15.5%)	23 (10.6%)	31 (13.4%)	54 (12.0%)	125 (18.0%)	101 (14.5%)	226 (16.3%)
F30	Banana	40 (15.7%)	49 (16.8%)	89 (16.4%)	95 (18.7%)	77 (16.8%)	172 (17.8%)	21 (9.7%)	26 (11.2%)	47 (10.5%)	87 (12.5%)	97 (14%)	184 (13.2%)
**Seasonal Fruits:**													

Table S3: Percentage Consumption of Selected Foods in Children aged 9-12 years and 13-16 years (cont)

ACAES FFQ Item		9 – 12 year old participants						13 – 16 year old participants					
		NNS (1995)			ANCNPAS (2007)			NNS (1995)			ANCNPAS (2007)		
		Female n=253	Male n=291	Persons n=544	Female n=509	Male n=457	Persons n=966	Female n=217	Male n=232	Persons n=449	Female n=694	Male n=695	Persons n=1389
FS1	Peach, nectarine, plum or apricot	24 (9.4%)	16 (5.5%)	40 (7.4%)	14 (2.8%)	14 (3.1%)	28 (2.9%)	22 (10.1%)	6 (2.6%)	28 (6.2%)	21 (3.0%)	9 (1.3%)	30 (2.2%)
FS2	Mango or Paw-paw	0 (0%)	3 (1.0%)	3 (0.6%)	0 (0%)	7 (1.5%)	7 (0.7%)	0 (0%)	1 (0.4%)	1 (0.2%)	0 (0%)	2 (0.3%)	2 (0.1%)
FS3	Pineapple	16 (6.3%)	14 (4.8%)	30 (5.550	9 (1.8%)	6 (1.3%)	15 (1.6%)	5 (2.3%)	8 (3.4%)	13 (2.9%)	11 (1.6%)	8 (1.2%)	19 (1.4%)
FS4	Grapes, strawberries, blueberries	22 (8.7%)	24 (8.2%)	46 (8.5%)	46 (9.0%)	38 (8.3%)	84 (8.7%)	11 (5.1%)	13 (5.6%)	24 (5.3%)	57 (8.2%)	40 (5.8%)	97 (7%)
FS5	Melon	14 (5.5%)	12 (4.1%)	26 (4.8%)	29 (5.7%)	23 (5%)	52 (5.4%)	7 (3.2%)	8 (3.4%)	15 (3.3%)	25 (3.6%)	20 (2.9%)	45 (3.2%)

Table S4: Changes to portion size (grams) between 1995 and 2007 for 2-4 year old children

	Females						Males						Persons					
	1995		2007				1995		2007				1995		2007			
Food	n =	Median [IQR]	n =	Median [IQR]	Δ Median	p-value	n =	Median [IQR]	n =	Median [IQR]	Δ Median	p-value	n =	Median [IQR]	n =	Median [IQR]	Δ Median	p-value
**Drinks**																		
Soft Drink	96	197 [130]	89	152 [105]	-45	0.002	118	203 [184]	97	155 [105]	-48	<0.001	214	197 [147]	186	153 [105]	-44	<0.001
Water	233	500 [500]	1686	180 [150]	-320	<0.001	214	500 [500]	1803	200 [140]	-300	<0.001	447	500 [500]	3489	188 [150]	-312	<0.001
Fruit Juice	160	193 [131]	492	131 [105]	-62	<0.001	180	193 [131]	520	131 [150]	-62	<0.001	340	193 [131]	1012	131 [114]	-62	<0.001
**Dairy Foods**																		
Whole Milk	451	186 [129]	1034	155 [119]	-31	0.002	455	194 [129]	1183	165 [133]	-29	<0.001	906	194 [129]	2217	155 [129]	-39	<0.001
Reduced Fat Milk	50	130 [141]	219	130 [102]	0	0.04	38	259 [130]	221	134 [103]	-125	<0.001	88	195 [130]	440	130 [103]	-65	<0.001
Flavored Milk	27	250 [155]	68	183 [130]	-67	0.02	16	258 [52]	80	208 [103]	-50	0.16	43	251 [138]	148	206 [129]	-45	0.01
Ice cream	62	55 [55]	126	42 [38]	-13	0.004	57	62 [91]	109	48 [38]	-14	<0.001	119	62 [65]	235	47 [38]	-15	<0.001
Yoghurt	42	125 [115]	299	100 [50]	-25	0.03	41	104 [130]	315	100 [60]	-4	0.60	83	110 [115]	614	100 [60]	-10	0.06
Cheese	129	20 [10]	440	20 [12]	0	0.17	144	20 [9]	484	21 [13]	1	0.04	273	20 [10]	924	20 [12]	0	0.02
Cheese Spread	32	19 [6]	47	10 [15]	-9	0.001	28	18 [6]	48	10 [15]	-8	<0.001	60	18 [6]	95	10 [15]	-8	<0.001
**Breads & Cereals**																		
Hot Porridge	15	195 [130]	56	133 [212]	-62	0.13	9	260 [98]	57	195 [194]	-65	0.005	24	260 [89]	113	190 [204]	-70	0.006
Breakfast Cereal	205	30 [15]	462	23 [17]	-7	0.10	222	30 [26]	521	30 [18]	0	0.14	427	30 [20]	983	26 [18]	-4	0.03
White Bread	317	48 [37]	494	37 [33]	-11	0.007	354	54 [32]	589	42 [34]	-12	0.001	671	49 [32]	1083	40 [34]	-9	<0.001
Mixed Grain Bread	36	52 [39]	109	42 [34]	-10	0.49	29	68 [37]	153	48 [34]	-20	0.11	65	52 [40]	262	48 [34]	-4	0.63
Wholemeal Bread	79	48 [37]	255	40 [32]	-8	0.65	67	48 [32]	246	48 [32]	0	0.60	146	48 [33]	501	42 [33]	-6	0.96
Rice	33	95 [127]	128	95 [94]	0	0.40	36	95 [142]	122	95 [141]	0	0.86	69	95 [140]	250	95 [97]	0	0.47
Pasta	38	148 [75]	229	113 [111]	-35	0.92	43	148 [148]	247	120 [120]	-28	0.32	81	148 [79]	476	113 [114]	-35	0.53
**Sweets & Snacks**																		
Cake	87	50 [48]	190	45 [39]	-5	0.04	62	43 [40]	218	49 [47]	6	0.31	149	49 [50]	408	47 [42]	-2	0.41

Table S4: Changes to portion size (grams) between 1995 and 2007 for 2-4 year old children (cont)

	Females						Males						Persons					
	1995		2007				1995		2007				1995		2007			
Food	n =	Median [IQR]	n =	Median [IQR]	Δ Median	p-value	n =	Median [IQR]	n =	Median [IQR]	Δ Median	p-value	n =	Median [IQR]	n =	Median [IQR]	Δ Median	p-value
Other desserts	57	100 [90]	66	86 [64]	-14	0.59	50	120 [90]	87	140 [51]	20	0.83	107	114 [90]	153	140 [58]	26	0.51
Plain sweet biscuits	84	18 [16]	171	16 [12]	-2	0.36	90	19 [20]	187	16 [14]	-3	0.21	174	18 [19]	358	16 [14]	-2	0.11
Cream/chocolate biscuits	51	17 [16]	80	19 [11]	2	0.57	44	21 [19]	102	18 [10]	-3	0.08	95	19 [16]	182	19 [10]	0	0.42
Savoury Biscuits	90	12 [13]	330	13 [11]	1	0.44	79	13 [9]	332	13 [12]	0	0.26	169	13 [11]	662	13 [12]	0	0.81
Muesli Bar	21	31 [3]	57	31 [12]	0	0.98	39	31 [3]	62	31 [11]	0	0.98	60	31 [3]	119	31 [11]	0	0.91
**Main Meals**																		
Mince Dish	18	63 [90]	125	70 [52]	7	0.81	26	74 [85]	123	59 [87]	-15	0.37	44	74 [87]	248	69 [66]	-5	0.38
Plain Meat	47	52 [56]	122	46 [41]	-6	0.41	52	46 [41]	125	47 [49]	1	0.77	99	47 [45]	247	46 [50]	-1	0.44
Crumbed Chicken	19	67 [74]	73	56 [51]	-11	0.94	16	69 [51]	50	62 [56]	-7	0.40	35	67 [61]	123	56 [54]	-11	0.56
Plain Chicken	31	55 [38]	121	49 [52]	-6	0.95	31	48 [3]4	134	60 [53]	12	0.13	62	49 [35]	255	57 [51]	8	0.26
Fish	39	48 [40]	104	71 [54]	23	<0.001	27	49 [28]	80	71 [56]	22	0.03	66	48 [33]	184	71 [64]	23	<0.001
Soup	21	255 [175]	50	193 [172]	-62	0.14	21	258 [247]	57	229 [183]	-29	0.67	42	257 [189]	107	195 [175]	-62	0.19
Sausages	50	25 [31]	96	41 [30]	16	0.21	46	55 [41]	110	44 [39]	-11	0.17	96	51 [36]	206	41 [28]	-10	0.08
Pie	37	87 [73]	32	16 [65]	-71	0.74	22	97 [111]	58	98 [84]	1	0.44	59	87 [85]	90	97 [76]	10	0.63
**Other Foods**																		
Chips (not potato)	36	21 [11]	72	20 [15]	-1	0.09	41	22 [16]	80	18 [12]	-4	0.03	77	21 [13]	152	18 [13]	-3	0.005
Potato crisps	33	25 [16]	77	20 [7]	-5	0.02	64	25 [4]	71	21 [5]	-4	0.04	97	25 [6]	148	21 [6]	-4	<0.001
Chocolate	92	18 [18]	152	15 [10]	-3	0.03	91	19 [17]	176	14 [12]	-5	0.02	183	18 [18]	328	14 [10]	-4	0.002
Lollies	75	14 [11]	135	12 [12]	-2	0.29	76	14 [10]	122	12 [12]	-2	0.77	151	14 [12]	257	12 [12]	-2	0.36
Jam/Honey	51	7 [7]	211	7 [7]	0	0.17	53	7 [8]	260	7 [7]	0	0.15	104	7 [8]	471	7 [7]	0	0.03
Peanut Butter	86	9 [8]	110	7 [7]	-2	0.57	83	6 [14]	127	8 [8]	2	0.46	169	9 [13]	237	8 [8]	-1	0.35
Tomato Sauce	101	6 [16]	147	6 [16]	0	0.13	82	8 [16]	186	7 [16]	-1	0.55	183	6 [16]	333	6 [16]	0	0.11
Luncheon Meats	39	18 [27]	50	28 [33]	10	0.02	33	29 [40]	65	28 [37]	-1	0.92	72	26 [36]	115	28 [37]	2	0.10
Bacon/Ham	50	16 [18]	145	21 [25]	5	0.001	61	18 [18]	161	25 [20]	7	<0.05	111	17 [17]	306	24 [23]	7	<0.001

Table S4: Changes to portion size (grams) between 1995 and 2007 for 2-4 year old children (cont)

	Females						Males						Persons					
	1995		2007				1995		2007				1995		2007			
Food	n =	Median [IQR]	n =	Median [IQR]	Δ Median	p-value	n =	Median [IQR]	n =	Median [IQR]	Δ Median	p-value	n =	Median [IQR]	n =	Median [IQR]	Δ Median	p-value
Eggs	41	49 [23]	108	43 [38]	-6	0.92	32	49 [19]	106	42 [41]	-7	0.33	73	49 [14]	214	43 [40]	-6	0.47
**Fruit and Vegetables**																		
Hot Chips	72	74 [65]	118	75 [60]	1	0.19	74	90 [44]	120	75 [55]	-15	0.001	146	90 [50]	238	75 [60]	-15	0.002
Potato	99	61 [84]	187	56 [58]	-5	0.07	89	65 [92]	209	61 [70]	-4	0.10	188	63 [89]	396	61 [57]	-2	0.02
Pumpkin	21	66 [66]	64	47 [50]	-19	0.28	26	53 [49]	60	30 [48]	-23	0.14	47	57 [66]	124	41 [44]	-16	0.10
Cauliflower	11	33 [55]	50	34 [29]	1	0.36	5	27 [35]	43	29 [16]	2	0.51	16	33 [47]	93	29 [29]	-4	0.40
Green Beans	31	19 [21]	53	25 [25]	6	0.05	21	20 [29]	61	22 [19]	2	0.67	52	19 [25]	114	25 [20]	6	0.10
Peas	48	26 [28]	113	12 [28]	-14	0.02	51	24 [27]	140	16 [15]	-8	0.01	99	26 [27]	253	14 [24]	-12	<0.001
Broccoli	25	23 [30]	116	22 [22]	-1	0.56	21	41 [60]	107	20 [23]	-21	0.007	47	29 [41]	223	20 [22]	-9	0.13
Carrots	98	23 [32]	255	18 [22]	-5	0.33	98	19 [30]	298	17 [23]	-2	0.10	196	21 [38]	553	17 [22]	-4	0.06
Eggplant/Zucchini	13	21 [19]	39	18 [16]	-3	0.87	10	10 [12]	54	22 [25]	12	<0.05	23	15 [16]	93	19 [24]	4	0.16
Capsicum	10	14 [15]	42	12 [16]	-2	0.91	19	5 [22]	55	11 [17]	6	0.08	29	6 [18]	97	11 [17]	5	0.14
Corn	32	34 [49]	106	23 [49]	-11	0.36	16	30 [71]	119	29 [30]	-1	0.37	48	32 [66]	225	27 [30]	-5	0.21
Mushroom	17	13 [13]	40	14 [17]	1	0.52	20	14 [21]	45	13 [17]	-1	0.69	37	13 [19]	85	13 [17]	0	0.90
Tomato	42	31 [31]	135	34 [44]	3	0.37	33	30 [69]	131	45 [37]	15	0.25	75	30 [40]	266	40 [41]	10	0.10
Lettuce	35	10 [11]	49	8 [15]	-2	0.53	28	9 [9]	34	7 [6]	-2	0.02	63	9 [11]	83	8 [10]	-1	0.07
Celery/Cucumber	26	19 [27]	86	20 [34]	1	0.30	31	11 [20]	81	20 [23]	9	0.15	57	13 [22]	167	20 [23]	7	0.06
Onion	12	9 [35]	106	8 [8]	-1	0.19	9	4 [18]	131	8 [9]	4	0.40	21	9 [20]	237	8 [9]	-1	0.65
Canned Fruit	6	130 [54]	52	80 [66]	-50	0.07	2	102 [77]	60	72 [81]	-30	0.38	8	130 [67]	112	80 [78]	-50	0.04
Dried Fruit	39	14 [23]	132	20 [30]	6	0.36	31	22 [35]	167	19 [30]	-3	0.22	70	16 [36]	299	20 [30]	4	0.89
Apple	162	85 [84]	453	83 [100]	-2	0.28	186	112 [70]	509	87 [92]	-25	0.05	348	106 [79]	962	83 [95]	-23	0.04
Orange	77	72 [58]	186	66 [52]	-6	0.06	56	86 [83]	208	86 [49]	0	0.58	133	86 [73]	394	72 [53]	-14	0.12
Banana	92	77 [50]	319	94 [74]	17	0.05	91	90 [51]	302	94 [61]	4	0.04	183	83 [51]	621	94 [6]0	11	0.003
**Seasonal Fruit**																		

Table S4: Changes to portion size (grams) between 1995 and 2007 for 2-4 year old children (cont)

	Females						Males						Persons					
	1995		2007				1995		2007				1995		2007			
Food	n =	Median [IQR]	n =	Median [IQR]	Δ Median	p-value	n =	Median [IQR]	n =	Median [IQR]	Δ Median	p-value	n =	Median [IQR]	n =	Median [IQR]	Δ Median	p-value
Mixed Berries/Grapes	33	45 [68]	187	50 [55]	5	0.38	25	83 [93]	177	56 [52]	-27	0.01	58	72 [57]	364	50 [50]	-22	0.40
Melon	19	150 [153]	84	75 [124]	-75	0.05	11	285 [406]	106	111 [140]	-174	0.04	30	161 [255]	190	87 [117]	-74	0.02

Table S5: Changes to portion size (grams) between 1995 and 2007 for 5-8 year old children

	Females						Males						Persons					
Food	n =	1995 Median [IQR]	n =	2007 Median [IQR]	Δ Median	p-.value	n =	1995 Median [IQR]	n =	2007 Median [IQR]	Δ Median	p-value	n =	1995 Median [IQR]	n =	2007 Median [IQR]	Δ Median	p-value
**Drinks**																		
Soft Drink	123	261 [117]	117	234 [148]	-27	0.10	207	262 [181]	129	293 [190]	31	0.71	330	261 [194]	246	261 [171]	0	0.08
Water	303	500 [500]	1087	230 [195]	-270	<0.001	313	500 [750]	1076	250 [250]	-250	<0.001	616	500 [624]	2163	250 [245]	-250	<0.001
Fruit Juice	142	210 [132]	282	210 [131]	0	0.01	169	210 [131]	345	241 [131]	31	0.47	311	210 [131]	627	210 [131]	0	0.02
Tea/Coffee	34	171 [127]	33	180 [117]	9	0.10	34	254 [127]	38	233 [72]	-21	0.09	68	254 [127]	71	200 [115]	-54	0.02
**Dairy Food**																		
Whole Milk	198	194 [129]	376	194 [129]	0	0.08	240	258 [129]	418	206 [129]	-52	0.06	438	217 [129]	196	200 [129]	-17	0.008
Reduced Fat Milk	55	194 [130]	124	155 [108]	-39	0.02	41	260 [130]	147	194 [129]	-66	<0.001	96	195 [130]	271	170 [129]	-25	<0.001
Flavored Milk	23	258 [102]	81	226 [72]	-32	0.11	42	260 [52]	63	256 [124]	-4	0.10	65	258 [67]	144	250 [93]	-8	0.006
Ice cream	65	93 [66]	123	56 [81]	-37	<0.001	73	102 [113]	114	62 [73]	-40	<0.001	138	96 [76]	237	60 [75]	-36	<0.001
Yoghurt	28	150 [136]	107	100 [80]	-50	0.08	20	142 [93]	121	100 [80]	-42	0.04	48	150 [120]	228	100 [80]	-50	0.01
Cheese	114	20 [12]	223	21 [20]	1	0.004	112	20 [19]	259	21 [11]	1	0.04	226	20 [17]	482	21 [16]	1	<0.001
**Breads and Cereals**																		
Breakfast Cereal	213	30 [23]	257	20 [21]	-10	0.38	251	38 [15]	299	33 [18]	-5	0.44	464	30 [15]	556	30 [15]	0	0.94
White Bread	399	54 [32]	399	56 [27]	2	0.15	436	64 [24]	394	62 [18]	-2	0.91	835	54 [32]	793	60 [27]	6	0.44
Mixed Grain Bread	31	56 [34]	67	62 [34]	6	0.92	40	42 [28]	52	64 [31]	22	<0.001	71	52 [40]	119	64 [28]	12	0.009
Wholemeal Bread	51	54 [32]	115	57 [24]	3	0.54	76	57 [32]	151	60 [26]	3	0.31	127	54 [32]	266	60 [25]	6	0.25
Rice	28	122 [170]	73	111 [101]	-11	0.79	53	190 [154]	64	162 [158]	-28	0.70	81	190 [150]	137	144 [114]	-46	0.32
Pasta	45	148 [148]	102	150 [115]	2	0.49	32	148 [145]	128	150 [150]	2	0.72	77	148 [125]	230	150 [133]	2	0.75
Cake	101	55 [45]	177	53 [51]	-2	0.94	132	66 [79]	145	53 [50]	-13	0.08	233	59 [72]	322	53 [50]	-6	0.15
**Sweets and**																		

Table S5: Changes to portion size (grams) between 1995 and 2007 for 5-8 year old children (cont)

	Females						Males						Persons					
Food	n =	1995 Median [IQR]	n =	2007 Median [IQR]	Δ Median	p-.value	n =	1995 Median [IQR]	n =	2007 Median [IQR]	Δ Median	p-value	n =	1995 Median [IQR]	n =	2007 Median [IQR]	Δ Median	p-value
**Snacks**																		
Sweet pie/pastry	34	59 [97]	27	50 [43]	-9	0.70	24	75 [73]	30	60 [29]	-15	0.25	58	68 [72]	57	60 [36]	-8	0.41
Other desserts	38	140 [140]	42	145 [92]	5	0.82	48	140 [59]	41	150 [50]	10	0.55	86	140 [61]	83	150 [50]	10	0.56
Plain sweet biscuits	98	16 [15]	102	22 [12]	6	0.01	133	20 [23]	103	24 [19]	4	0.13	231	18 [17]	205	22 [15]	4	0.01
Cream/chocolate biscuits	80	24 [23]	76	20 [13]	-4	0.08	76	21 [23]	87	21 [19]	0	0.67	156	23 [24]	163	20 [15]	-3	0.35
Savoury Biscuits	101	15 [15]	183	18 [15]	3	0.47	113	20 [14]	174	20 [13]	0	0.96	214	18 14]	357	18 [13]	0	0.71
Muesli Bar	49	31 [2]	59	31 [13]	0	0.20	45	31 [3]	66	31 [11]	0	<0.05	94	31 [2]	125	31 [11]	0	0.03
**Main Meals**																		
Mince Dish	37	80 [90]	54	78 [99]	-2	0.96	42	124 [96]	85	85 [102]	-39	0.24	79	97 [88]	139	85 [96]	-12	0.45
Plain Meat	66	49 [37]	69	56 [50]	7	0.09	68	56 [53]	81	67 [59]	11	0.40	134	52 [46]	150	65 [53]	13	0.07
Crumbed Chicken	29	72 [71]	59	66 [36]	-6	0.83	39	72 [54]	56	82 [62]	10	0.87	68	72 [61]	115	72 [54]	0	0.95
Plain Chicken	56	45 [61]	86	67 [71]	22	0.12	65	70 [56]	87	76 [85]	6	0.07	121	67 [64]	173	72 [80]	5	0.04
Fish	39	60 [72]	61	71 [67]	11	0.01	32	50 [59]	46	74 [87]	24	0.008	71	58 [64]	107	71 [70]	13	<0.001
Soup	19	253 [187]	36	300 [259]	47	0.72	35	255 [11]	30	253 [237]	-2	0.30	54	255 [87]	66	259 [256]	4	0.76
Sausages	54	57 [33]	65	57 [30]	0	0.03	63	69 [52]	78	58 [38]	-11	0.10	116	59 [48]	143	58 [39]	-1	0.009
Pizza	27	72 [72]	37	116 [87]	44	0.01	46	115 [103]	31	130 [134]	15	0.38	73	100 [88]	68	123 [101]	23	0.07
Pie	46	112 [87]	56	124 [96]	12	0.40	43	174 [174]	44	140 [58]	-34	0.63	89	131 [85]	100	130 [64]	-1	0.77
**Other Foods**																		
Chips (not potato)	59	22 [16]	52	20 [23]	-2	0.95	70	21 [8]	66	25 [25]	4	0.14	129	21 [9]	118	25 [24]	4	0.33
Potato crisps	92	25 [4]	96	21 [1]	-4	<0.001	93	25 [7]	106	21 [30]	-4	0.52	185	25 [7]	202	21 [7]	-4	<0.001
Ice block - water	42	73 [5]	42	74 [7]	1	0.41	57	74 [5]	36	75 [8]	1	0.19	99	74 [5]	78	75 [6]	1	0.14
Chocolate	158	20 [18]	166	20 [23]	0	0.64	132	19 [23]	159	20 [28]	1	0.57	290	20 [19]	325	20 [25]	0	0.46
Lollies	119	15 [16]	131	14 [16]	-1	0.87	118	13 [19]	127	16 [14]	3	0.12	237	14 [16]	258	15 [16]	1	0.19

Table S5: Changes to portion size (grams) between 1995 and 2007 for 5-8 year old children (cont)

	Females						Males						Persons					
Food	n =	1995 Median [IQR]	n =	2007 Median [IQR]	Δ Median	p-.value	n =	1995 Median [IQR]	n =	2007 Median [IQR]	Δ Median	p-value	n =	1995 Median [IQR]	n =	2007 Median [IQR]	Δ Median	p-value
Salad Dressing/Mayo full fat	28	4 [18]	22	5 [13]	1	0.72	21	7 [7]	26	9 [15]	2	0.65	49	5 [14]	48	6 [14]	1	0.57
Jam/Honey	51	7 [6]	124	12 [7]	5	0.22	74	10 [6]	122	8 [7]	-2	0.46	125	7 [7]	246	11 [7]	4	0.19
Peanut Butter	82	13 [15]	84	10 [13]	-3	0.95	102	9 [13]	96	10 [13]	1	0.41	184	10 [15]	180	10 [13]	0	0.66
Tomato Sauce	120	8 [16]	148	11 [16]	3	0.07	149	8 [16]	145	13 [16]	5	0.008	269	8 [16]	293	11 [16]	3	0.002
Luncheon Meats	39	17 [15]	56	28 [21]	11	0.003	32	24 [63]	43	28 [36]	4	0.83	71	18 [27]	99	28 [37]	10	0.02
Bacon/Ham	60	25 [21]	107	34 [26]	9	<0.001	72	22 [26]	128	29 [30]	7	0.01	132	24 [21]	235	30 [26]	6	<0.001
Eggs	36	50 [23]	64	44 [36]	-6	0.28	47	50 [59]	76	43 [52]	-7	0.14	83	50 [47]	140	43 [45]	-7	0.06
**Fruit and Vegetables**																		
Hot Chips	63	100 [60]	108	75 [40]	-25	<0.001	113	100 [69]	97	77 [38]	-23	0.002	176	100 [62]	205	75 [40]	-25	<0.001
Potato	104	116 [74]	111	75 [73]	-41	0.003	102	91 [86]	106	92 [78]	1	0.41	206	93 [79]	217	75 [76]	-18	0.008
Pumpkin	28	65 [86]	25	54 [59]	-11	0.39	20	50 [45]	27	49 [69]	-1	0.87	48	65 [56]	52	60 [54]	-5	0.49
Cauliflower	15	33 [55]	24	34 [29]	1	0.30	12	33 [16]	27	29 [29]	-4	0.99	27	33 [17]	51	29 [29]	-4	0.38
Green Beans	32	35 [44]	37	26 [17]	-9	0.06	30	31 [36]	33	32 [29]	1	0.66	62	35 [41]	64	26 [22]	-9	0.10
Peas	54	40 [40]	61	20 [29]	-20	0.04	50	40 [43]	56	22 [29]	-18	0.008	104	40 [39]	117	20 [29]	-20	0.001
Broccoli	27	23 [38]	63	26 [21]	3	0.89	30	45 [56]	67	26 [33]	-19	0.03	57	34 [49]	63	26 [21]	-8	0.13
Carrots	120	35 [31]	166	29 [28]	-6	0.04	113	31 [37]	160	30 [37]	-1	0.88	233	32 [34]	326	29 [33]	-3	0.17
Capsicum	23	19 [25]	39	19 [17]	0	0.80	21	11 [19]	25	14 [10]	3	0.81	44	14 [16]	64	17 [15]	3	0.85
Corn	23	44 [45]	57	24 [48]	-20	0.11	30	51 [53]	48	35 [37]	-16	0.07	53	49 [47]	105	32 [44]	-17	0.01
Tomato	75	48 [38]	77	60 [46]	12	0.20	49	45 [36]	82	47 [50]	2	0.37	124	46 [38]	159	51 [49]	5	0.19
Lettuce	66	14 [12]	59	8 [9]	-6	0.32	63	15 [14]	72	11 [20]	-4	0.82	129	15 [12]	131	9 [13]	-6	0.44
Celery/Cucumber	53	21 [17]	71	25 [18]	4	0.38	39	25 [30]	73	20 [30]	-5	0.69	92	21 [23]	144	24 [24]	3	0.91
Onion	17	15 [27]	64	9 [12]	-6	0.29	35	14 [22]	67	9 [14]	-5	0.32	52	15 [24]	131	9 [12]	-6	0.13
Canned Fruit	5	135 [29]	26	104 [95]	-31	0.19	2	100 [80]	35	78 [84]	-22	0.50	7	135 [47]	61	93 [83]	-42	0.05

Table S5: Changes to portion size (grams) between 1995 and 2007 for 5-8 year old children (cont)

	Females						Males						Persons					
Food	n =	1995 Median [IQR]	n =	2007 Median [IQR]	Δ Median	p-.value	n =	1995 Median [IQR]	n =	2007 Median [IQR]	Δ Median	p-value	n =	1995 Median [IQR]	n =	2007 Median [IQR]	Δ Median	p-value
Dried Fruit	30	21 [29]	30	27 [23]	6	0.99	16	43 [42]	36	21 [39]	-22	0.16	46	29 [30]	66	25 [31]	-4	0.21
Apple	166	140 [59]	238	156 [72]	16	0.24	155	140 [26]	224	166 [58]	26	0.11	321	140 [41]	462	161 [58]	21	<0.05
Orange	70	101 [45]	116	86 [45]	-15	0.41	77	131 [58]	98	86 [59]	-45	0.03	147	101 [45]	214	86 [45]	-15	0.04
Banana	77	96 [51]	125	121 [27]	25	<0.001	79	96 [26]	129	121 [27]	25	<0.001	156	96 [31]	254	121 [27]	25	<0.001
**Seasonal Fruit**																		
Mixed Berries/Grapes	34	63 [49]	73	60 [71]	-3	0.44	24	73 [122]	63	56 [49]	-17	0.11	58	63 [72]	136	60 [78]	-3	0.59
Melon	15	151 [92]	55	133 [113]	-18	0.80	18	83 [80]	48	150 [217]	67	0.14	33	85 [106]	55	133 [113]	48	0.25

Table S6: Changes to portion size (grams) between 1995 and 2007 for 9-12 year old children

	Females						Males						Persons					
	1995		2007				1995		2007				1995		2007			
Food	n =	Median [IQR]	n =	Median [IQR]	Δ Median	p-value	n =	Median [IQR]	n =	Median [IQR]	Δ Median	p-value	n =	Median [IQR]	n =	Median [IQR]	Δ Median	p-value
**Drinks**																		
Diet Soft Drink	25	250 [125]	32	278 [260]	28	0.90	36	300 [175]	31	300 [145]	0	0.57	61	281 [140]	63	280 [175]	-1	0.65
Soft Drink	176	313 [132]	194	263 [182]	-50	0.02	198	391 [187]	209	245 [195]	-146	0.26	374	327 [156]	403	304 [182]	-23	0.01
Water	287	700[795]	1259	250 [228]	-450	<0.001	310	750 [879]	1110	250 [250]	-500	<0.001	597	750 [850]	2369	250 [235]	-500	<0.001
Fruit Juice	129	263 [74]	322	263 [105]	0	<0.001	118	262 [131]	266	261 [52]	-1	0.43	247	262 [107]	588	241 [62]	-21	0.006
Tea/Coffee	119	254 [92]	89	240 [47]	-14	<0.001	42	254 [80]	32	240 [62]	-14	0.007	77	254 [92]	57	240 36]	-14	0.003
**Dairy Foods**																		
Whole Milk	277	206 [140]	322	232 [129]	26	0.86	328	258 [194]	346	257 [257]	-1	0.001	605	258 [129]	668	237 [129]	-21	0.01
Reduced Fat Milk	111	195 [130]	174	194 [172]	-1	0.02	119	260 [130]	177	237 [131]	-23	0.03	230	259 [130]	351	206 [129]	-53	0.001
Skim Milk	17	259 [162]	53	194 [211]	-65	0.11	15	259 [169]	27	237 [193]	-22	<0.05	32	259 [129]	80	194 [199]	-65	0.005
Flavored Milk	24	307 [54]	88	258 [145]	-49	0.56	41	292 [123]	137	258 [119]	-34	0.22	65	304 [92]	225	258 [124]	-46	0.18
Cream/Sour Cream	24	20 [31]	26	23 [30]	3	0.39	18	19 [19]	28	20 [28]	1	0.58	42	20 [32]	54	20 [30]	0	0.35
Ice cream	83	100 [110]	124	100 [74]	0	0.58	97	124 [125]	107	90 [174]	-34	0.04	180	124 [126]	231	93 [74]	-31	0.06
Yoghurt	25	207 [87]	78	100 [107]	-107	<0.001	24	200 [142]	77	100 [100]	-100	0.002	49	200 [120]	155	100 [100]	-100	<0.001
Cheese	135	20 [19]	252	21 [15]	1	0.007	129	21 [19]	203	21 [21]	0	0.13	264	21 [17]	455	21 [20]	0	0.003
**Breads and Cereal**																		
Cooked Porridge	6	336 [531]	23	130 [260]	-206	0.06	4	311 [332]	26	358 [218]	47	0.88	10	336 [423]	49	260 [281]	-76	0.21
Breakfast Cereal	160	38 [30]	199	41 [30]	3	0.88	243	60 [32]	271	45 [36]	-15	0.02	403	45 [38]	470	45 [30]	0	0.05
White Bread	402	57 [32]	400	62 [24]	5	0.36	429	64 [26]	402	64 [26]	0	0.02	831	64 [28]	802	64 [32]	0	0.23

Table S6: Changes to portion size (grams) between 1995 and 2007 for 9-12 year old children (cont)

	Females						Males						Persons					
	1995		2007				1995		2007				1995		2007			
Food	n =	Median [IQR]	n =	Median [IQR]	Δ Median	p-value	n =	Median [IQR]	n =	Median [IQR]	Δ Median	p-value	n =	Median [IQR]	n =	Median [IQR]	Δ Median	p-value
Mixed Grain Bread	27	56 [17]	66	64 [25]	8	0.52	24	68 [19]	43	66 [29]	-2	0.76	51	68 [17]	109	64 [27]	-4	0.80
Wholemeal Bread	51	64 [16]	97	64 [28]	0	0.74	70	64 [20]	99	64 [16]	0	0.18	121	64 [12]	196	64 [24]	0	0.21
Rice	39	190 [160]	86	158 [121]	-32	0.01	36	190 [226]	73	190 [165]	0	0.23	75	190 [190]	159	190 [125]	0	0.008
Pasta	24	230 [148]	134	150 [146]	-80	0.14	31	261 [200]	131	225 [196]	-36	0.56	55	237 [158]	265	188 [173]	-49	0.14
**Sweets and Snacks**																		
Cake	105	61 [67]	195	63 [70]	2	0.24	116	66 [65]	166	82 [102]	16	0.05	221	64 [68]	361	72 [85]	8	0.05
Sweet pie/pastry	36	86 [105]	52	63 [57]	-23	<0.05	24	71 [96]	42	80 [74]	9	0.93	60	82 [99]	94	75 [64]	-7	0.10
Other desserts	28	140 [82]	72	133 [82]	-7	0.95	34	150 [165]	61	150 [280]	0	0.81	133	150 [107]	133	143 [160]	-7	0.88
Plain sweet biscuits	75	22 [22]	104	20 [17]	-2	0.22	76	23 [21]	75	25 [22]	2	0.23	151	22 [24]	179	22 [18]	0	0.82
Cream/chocolate biscuits	53	21 [22]	100	30 [30]	9	0.007	80	31 [28]	112	30 [50]	-1	0.38	133	27 [24]	212	30 [35]	3	0.03
Savoury Biscuits	72	21 [23]	169	18 [13]	-3	0.40	81	20 [20]	127	24 [12]	4	0.63	153	20 [20]	296	20 [13]	0	0.66
Muesli Bar	26	31 [1]	71	31 [11]	0	0.79	35	31 [2]	72	31 [11]	0	0.36	61	31 [2]	143	31 [11]	0	0.63
**Main Meals**																		
Mince Dish	37	89 [111]	88	129 [135]	40	0.22	43	100 [127]	97	140 [205]	40	0.09	80	96 [117]	185	138 [185]	42	0.04
Plain Meat	85	65 [62]	111	93 [98]	28	0.02	93	77 [73]	121	92 [138]	15	0.003	178	70 [66]	232	93 [125]	23	<0.001
Mixed chicken dish	11	200 [290]	30	100 [163]	-100	0.18	10	229 [189]	29	127 [172]	-102	0.06	21	206 [237]	59	108 [108]	-98	0.02
Crumbed Chicken	18	72 [59]	84	89 [98]	17	0.33	33	85 [61]	69	143 [211]	58	0.001	51	72 [56]	153	109 [233]	37	0.01
Plain Chicken	64	67 [82]	99	78 [78]	11	0.13	43	72 [100]	95	95 [85]	23	0.16	107	68 [82]	194	84 [90]	16	0.03
Fish	13	113 [75]	45	100 [73]	-13	0.12	24	96 [116]	41	108 [80]	12	0.41	37	99 [101]	86	104 [74]	5	0.78
Soup	24	306 [253]	68	260 [260]	-46	0.07	35	313 [315]	57	286 [307]	-27	0.38	59	313 [306]	125	274 [260]	-39	0.06

Table S6: Changes to portion size (grams) between 1995 and 2007 for 9-12 year old children (cont)

	Females						Males						Persons					
	1995		2007				1995		2007				1995		2007			
Food	n =	Median [IQR]	n =	Median [IQR]	Δ Median	p-value	n =	Median [IQR]	n =	Median [IQR]	Δ Median	p-value	n =	Median [IQR]	n =	Median [IQR]	Δ Median	p-value
Sausages	48	59 [52]	68	58 [73]	-1	0.48	72	63 [77]	71	72 [55]	9	0.71	120	61 [62]	139	58 [67]	-3	0.40
Hamburger	24	122 [86]	45	136 [110]	14	0.93	21	204 [113]	58	220 [250]	16	0.20	45	159 [95]	103	196 [164]	37	0.25
Pizza	32	87 [143]	48	110 [97]	23	0.36	45	117 [143]	37	164 [155]	47	0.04	77	104 [133]	85	132 [134]	28	0.11
Pie	46	165 [70]	77	130 [82]	-35	0.68	43	175 [57]	90	174 [130]	-1	0.12	89	174 [64]	167	170 [103]	-4	0.39
**Other Foods**																		
Chips (not potato)	70	21 [14]	84	37 [60]	16	<0.001	53	25 [26]	86	39 [188]	14	0.42	123	21 [12]	170	37 [99]	16	<0.001
Potato crisps	76	25 [8]	107	21 [9]	-4	0.004	70	25 [3]	119	21 [21]	-4	0.04	146	25 [3]	226	21 [14]	-4	<0.001
Ice block – creamy	39	74 [20]	29	65 [8]	-9	0.007	39	82 [27]	30	68 [36]	-14	0.22	78	78 [19]	59	68 [50]	-10	0.007
Ice block - water	34	75 [32]	35	75 [8]	0	0.64	34	75 [80]	33	76 [10]	1	0.81	68	75 [62]	68	76 [6]	1	0.88
Chocolate	116	23 [23]	182	18 [24]	-5	0.15	125	23 [30]	171	20 [31]	-3	0.28	241	23 [28]	353	20 [27]	-3	0.07
Lollies	104	14 [17]	139	16 [23]	2	<0.05	90	15 [28]	131	16 [25]	1	0.43	194	15 [18]	270	16 [23]	1	0.04
Salad Dressing/Mayonnaise full fat	37	9 [15]	43	9 [15]	0	0.64	31	14 [14]	40	11 [15]	-3	0.70	68	9 [14]	83	10 [14]	1	0.57
Jam/Honey	47	7 [7]	104	12 [14]	5	0.04	82	14 [20]	89	14 [17]	0	0.17	129	10 [13]	193	13 [16]	3	0.03
Peanut Butter	62	11 [10]	99	13 [31]	2	0.006	76	11 [13]	112	19 [35]	8	<0.001	138	11 [13]	211	14 [32]	3	<0.001
Tomato Sauce	105	11 [16]	160	13 [16]	2	0.03	141	13 [16]	166	11 [16]	-2	0.39	246	11 [16]	326	13 [16]	2	0.05
Luncheon Meats	18	23 [26]	54	40 [92]	17	0.02	56	28 [25]	64	35 [245]	7	0.009	74	27 [25]	118	40 [190]	13	<0.001
Bacon/Ham	66	27 [24]	107	30 [30]	3	0.008	65	26 [19]	99	30 [25]	4	0.04	131	26 [18]	206	30 [26]	4	<0.001
Eggs	43	49 [20]	60	43 [39]	-6	0.94	51	50 [56]	67	50 [41]	0	0.995	94	49 [37]	127	49 [41]	0	0.93
**Fruit and Vegetables**																		
Hot Chips	81	100 [60]	114	100 [75]	0	0.17	104	150 [100]	103	110 [75]	-40	<0.001	185	128 [69]	217	104 [75]	-24	<0.001
Potato	118	123 [144]	131	107 [73]	-16	<0.001	129	127 [143]	118	110 [118]	-17	0.03	247	124 [135]	249	110 [84]	-14	<0.001

Table S6: Changes to portion size (grams) between 1995 and 2007 for 9-12 year old children (cont)

	Females						Males						Persons					
	1995		2007				1995		2007				1995		2007			
Food	n =	Median [IQR]	n =	Median [IQR]	Δ Median	p-value	n =	Median [IQR]	n =	Median [IQR]	Δ Median	p-value	n =	Median [IQR]	n =	Median [IQR]	Δ Median	p-value
Pumpkin	37	65 [91]	38	65 [78]	0	0.88	40	85 [94]	26	68 [89]	-17	0.66	77	84 [95]	64	65 [81]	-19	0.78
Cauliflower	17	55 [33]	28	44 [29]	-11	0.99	22	33 [31]	27	50 [40]	17	<0.05	39	33 [39]	55	45 [35]	12	0.12
Green Beans	31	42 [32]	52	32 [26]	-10	0.13	36	42 [48]	32	32 [47]	-10	0.79	67	42 [38]	84	32 [38]	-10	0.16
Peas	68	53 [54]	69	27 [40]	-26	0.003	62	53 [58]	60	34 [30]	-19	0.03	130	53 [55]	129	33 [35]	-20	<0.001
Broccoli	27	65 [77]	73	30 [24]	-35	0.01	39	49 [64]	57	30 [31]	-19	0.20	66	55 [77]	130	30 [30]	-25	0.007
Carrots	106	38 [37]	183	29 [26]	-9	0.08	106	39 [37]	145	29 [29]	-10	0.01	212	39 [36]	328	29 [27]	-10	0.002
Capsicum	27	17 [16]	44	21 [19]	4	0.54	23	12 [11]	43	20 [18]	8	0.02	50	13 [17]	87	20 [18]	7	<0.05
Corn	28	44 [50]	54	43 [43]	-1	0.82	25	43 [53]	51	44 [29]	1	0.24	53	44 [52]	105	43 [51]	-1	0.30
Tomato	78	46 [54]	129	60 [40]	14	0.10	82	58 [65]	97	60 [40]	2	0.87	160	48 [54]	226	60 [40]	12	0.31
Lettuce	100	15 [21]	106	15 [21]	0	0.85	82	15 [11]	74	16 [21]	1	0.09	182	15 [17]	180	15 [21]	0	0.22
Celery/Cucumber	58	28 [25]	82	25 [22]	-3	0.83	45	19 [21]	57	25 [20]	6	0.20	103	22 [24]	139	25 [21]	3	0.50
Onion	34	17 [25]	87	14 [14]	-3	0.61	39	22 [20]	81	14 [15]	-8	0.001	73	18 [25]	168	14 [16]	-4	0.007
Apple	147	140 [26]	244	166 [58]	26	0.34	149	159 [26]	178	166 [57]	7	0.52	296	140 [26]	422	166 [58]	26	0.32
Orange	46	116 [45]	104	86 [59]	-30	0.02	65	101 [45]	77	86 [52]	-15	0.31	111	101 [45]	181	86 [59]	-15	0.02
Banana	44	102 [38]	105	121 [27]	19	0.67	59	101 [32]	91	121 [27]	20	0.008	103	102 [30]	196	121 [27]	19	0.02
**Seasonal Fruit**																		
Mixed Berries/Grapes	27	79 [190]	58	83 [91]	4	0.48	27	86 [123]	44	84 [94]	-2	0.65	54	86 [136]	102	84 [85]	-2	0.40
Melon	20	136 [179]	33	161[174]	25	0.65	16	148 [155]	28	177 [241]	29	0.37	36	145 [177]	61	161 [211]	16	0.36

Table S7: Changes to portion size (grams) between 1995 and 2007 for 13-16 year old children

	Females						Males						Persons					
	1995		2007				1995		2007				1995		2007			
Food	n =	Median [IQR]	n =	Median [IQR]	Δ Median	p-value	n =	Median [IQR]	n =	Median [IQR]	Δ Median	p-value	n =	Median [IQR]	n =	Median [IQR]	Δ Median	p-value
**Drinks**																		
Diet Soft Drink	31	375 [125]	61	375 [138]	0	0.62	26	375 [133]	84	375 [75]	0	0.07	57	375 [215]	145	375 [125]	0	0.54
Soft Drink	148	313 [132]	301	340 [178]	27	0.38	243	391 [260]	397	391 [187]	0	0.09	391	391 [187]	698	391 [156]	0	0.43
Water	238	775 [750]	1594	300 [335]	-475	<0.001	229	750 [750]	1564	300 [370]	-450	<0.001	467	750 [750]	3158	300 [380]	-450	<0.001
Fruit Juice	122	263 [171]	425	262 [52]	-1	0.03	86	263 [162]	401	262 [81]	-1	0.29	208	263 [118]	826	262 [105]	-1	0.03
Tea or Coffee	104	254 [51]	193	240 [33]	-14	<0.001	98	254 [30]	160	260 [54]	6	0.85	202	254 [51]	353	240 [38]	-14	0.001
**Dairy Foods**																		
Whole Milk	209	129 [193]	357	206 [176]	77	0.02	266	258 [258]	553	258 [214]	0	0.78	475	258 [224]	910	258 [194]	0	<0.05
Reduced Fat Milk	83	194 [195]	196	175 [196]	-19	0.40	103	259 [275]	216	258 [231]	-1	0.85	186	222 [260]	412	237 [216]	15	050
Flavored Milk	27	304 [87]	138	278 [142]	-26	0.81	36	353 [317]	130	353 [267]	0	0.29	63	310 [257]	268	308 [244]	-2	0.27
Cream	20	21 [29]	56	15 [19]	-5	0.46	10	20 [32]	38	20 [31]	0	0.99	30	20 [29]	94	17 [27]	-3	0.57
Ice Cream	45	124 [111]	107	112 [112]	-12	0.83	62	178 [186]	136	112 [106]	-66	0.001	107	128 [176]	243	112 [108]	-16	<0.01
Yoghurt	25	200 [77]	108	150 [114]	-50	0.09	19	200 [159]	79	200 [159]	0	0.72	44	200 [77]	187	156 [100]	-44	0.14
Cheese	123	21 [22]	330	22 [25]	1	0.03	122	24 [23]	297	33 [25]	9	0.005	245	21 [26]	627	23 [25]	2	<0.001
**Breads & Cereals**																		
Breakfast Cereal	104	45 [30]	214	45 [38]	0	0.18	184	60 [45]	385	60 [45]	0	0.62	288	60 [50]	599	60 [40]	0	0.18
White Bread	237	64 [12]	468	64 [34]	0	0.97	316	64 [56]	582	66 [20]	2	0.17	553	64 [27]	1050	64 [21]	0	0.28
Wholemeal Bread	46	54 [26]	132	64 [31]	10	0.02	32	64 [58]	114	68 [16]	4	0.45	81	54 [29]	246	64 [26]	10	0.01
Mixed Grain Bread	39	56 [32]	110	68 [29]	12	0.03	22	68 [13]	84	68 [28]	0	0.04	61	56 [18]	194	68 [22]	12	0.002
English Muffins	12	59 [58]	36	63 [34]	4	0.22	11	60 [42]	43	94 [63]	34	0.006	23	60 [42]	79	67 [59]	7	0.003
Pasta	35	222 [148]	151	210 [168]	-12	0.83	23	306 [248]	162	253 [182]	-53	0.12	58	289 [189]	313	225 [152]	-64	0.45
Rice	24	190 [135]	117	190 [186]	0	0.72	26	293 [190]	125	247 [237]	-45	0.45	50	231 [192]	242	216 [224]	-15	0.39
**Sweets & Snacks**																		

Table S7: Changes to portion size (grams) between 1995 and 2007 for 13-16 year old children (cont)

	Females						Males						Persons					
	1995		2007				1995		2007				1995		2007			
Food	n =	Median [IQR]	n =	Median [IQR]	Δ Median	p-value	n =	Median [IQR]	n =	Median [IQR]	Δ Median	p-value	n =	Median [IQR]	n =	Median [IQR]	Δ Median	p-value
Cakes/Pikelets	77	63 [61]	210	72 [80]	9	0.03	56	87 [79]	193	80 [81]	-7	0.75	133	70 [69]	403	76 [80]	6	0.16
Sweet Pies/Pastries	28	100 [129]	50	67 [61]	-33	0.13	35	102 [149]	53	80 [70]	-22	0.40	63	102 [136]	103	75 [72]	-27	0.09
Other Puddings	13	140 [89]	48	115 [105]	-25	0.94	19	140 [93]	37	150 [130]	10	0.45	32	140 [90]	85	140 [105]	0	0.80
Plain Sweet Biscuits	32	33 [25]	104	22 [22]	-11	0.10	45	30 [25]	106	28 [24]	-2	0.55	77	32 [25]	210	24 [24]	-8	0.09
Chocolate Biscuits	41	34 [27]	89	23 [22]	-11	0.11	47	35 [24]	116	28 [21]	-7	0.01	88	35 [24]	205	25 [21]	-10	0.003
Dry Savoury Biscuits	57	20 [17]	154	24 [20]	4	0.11	43	24 [23]	127	25 [23]	1	0.47	100	22 [17]	281	25 [20]	3	0.09
Muesli Bars	15	31 [0]	112	31 [10]	0	0.83	28	31 [2]	123	31 [11]	0	0.89	43	31 [0]	235	31 [11]	0	0.99
**Mail Meals**																		
Mince Dish	40	143 [187]	88	85 [103]	-58	0.004	43	130 [140]	101	150 [166]	20	0.16	83	142 [178]	189	118.5 [140]	-23	0.43
Plain Meat	77	72 [68]	141	80 [78]	8	0.16	70	90 [92]	159	104 [98]	14	0.28	147	86 [82]	300	88 [84]	2	0.06
Chicken with Sauce	15	150 [126]	40	111 [133]	-39	0.07	9	216 [274]	58	200 [189]	-16	0.85	24	181 [158]	98	143 [185]	-38	0.46
Crumbed Chicken	24	92 [104]	61	99 [89]	7	0.81	42	85 [54]	77	143 [135]	58	<0.001	66	90 [78]	138	117 [130]	28	0.006
Plain Chicken	60	68 [84]	130	101 [86]	34	0.02	58	108 [77]	163	120 [108]	12	0.06	118	89 [98]	293	107 [100]	18	0.002
Soup	14	305 [259]	74	329 [199]	24	0.95	12	506 [370]	61	300 [268]	-206	0.21	26	380 [266]	135	320 [218]	-60	0.33
Sausage/ Frankfurt	22	59 [41]	59	58 [48]	-1	0.44	40	81 [80]	98	82 [57]	1	0.51	62	127 [66]	157	114 [57]	-13	0.34
Hamburger	28	159 [57]	46	191 [113]	32	0.15	30	190 [103]	42	203 [124]	13	0.74	58	159 [95]	88	201 [123]	42	0.54
Pizza	27	74 [88]	56	142 [166]	68	<0.001	41	100 [144]	87	225 [184]	125	<0.001	68	91 [116]	143	174 [193]	83	<0.001
Pie/Sausage Roll	34	164 [64]	75	140 [64]	-24	0.76	56	174 [75]	106	174 [60]	0	0.76	90	174 [36]	181	170 [60]	-4	0.59
**Other Foods**																		
Chips (not potato)	37	22 [33]	75	28 [30]	6	0.16	34	25 [37]	58	50 [40]	25	0.52	71	25 [30]	133	30 [36]	5	0.20
Potato Chips	44	25 [25]	145	21 [29]	-4	0.03	58	25 [25]	147	21 [29]	-4	0.09	102	25 [25]	292	21 [29]	-4	0.006
Chocolate	109	33 [37]	237	24 [29]	-9	0.13	79	41 [40]	177	30 [37]	-11	0.11	188	36 [36]	414	26 [34]	-10	0.03

Table S7: Changes to portion size (grams) between 1995 and 2007 for 13-16 year old children (cont)

	Females						Males						Persons					
	1995		2007				1995		2007				1995		2007			
Food	n =	Median [IQR]	n =	Median [IQR]	Δ Median	p-value	n =	Median [IQR]	n =	Median [IQR]	Δ Median	p-value	n =	Median [IQR]	n =	Median [IQR]	Δ Median	p-value
Lollies	47	13 [8]	176	15 [22]	2	0.87	36	26 [46]	149	28 {29]	2	0.80	83	15 [20]	325	18 [30]	3	0.73
Salad Dressing/Mayo (not low fat)	31	10 [16]	74	10 [15]	0	0.96	35	14 [12]	84	10 [15]	-4	0.80	66	14 [14]	158	10 [15]	-4	0.92
Jam/Honey	33	7 [7]	151	13 [19]	6	0.005	24	14 [18]	130	14 [18]	0	0.52	57	10 [11]	281	13 [19]	3	0.009
Peanut Butter	17	13 [14]	90	12 [13]	-1	0.94	33	13 [22]	115	19 [13]	6	0.70	50	13 [19]	205	13 [15]	0	0.97
Tomato Sauce	94	11 [16]	191	22 [17]	11	<0.001	135	22 [16]	253	22 [20]	0	0.01	229	13 [16]	444	22 [18]	9	<0.001
Luncheon Meat	24	21 [28]	40	36 [34]	15	0.14	26	47 [37]	57	38 [47]	-11	0.55	50	35 [42]	97	36 [34]	1	0.54
Bacon/Ham	50	23 [24]	160	27 [22]	4	0.03	74	37 [41]	175	40 [35]	3	0.18	124	34 [25]	335	36 [33]	2	<0.05
Eggs	22	49 [39]	108	43 [54]	-6	0.90	46	67 [58]	91	43 [49]	-24	0.01	68	50 [61]	199	43 [55]	-7	0.30
**Fruit and Vegetables**																		
Hot Chips	65	114 [50]	187	112 [75]	-2	0.75	93	150 [60]	168	144 [41]	-6	0.51	158	137 [50]	355	133 [51]	-4	0.19
Potato	101	123 [102]	165	111 [100]	-12	0.13	87	184 [139]	166	146 [124]	-38	0.05	188	138 [131]	331	122 [121]	-16	0.04
Pumpkin	32	86 [78]	51	77 [75]	-9	0.83	26	69 [180]	27	90 [110]	21	0.59	58	75 [81]	78	85 [75]	10	0.60
Green Beans	26	42 [47]	63	33 [34]	-9	0.96	22	70 [79]	60	49 [33]	-21	0.02	48	63 [53]	123	43 [34]	-20	0.15
Peas	39	40 [53]	81	40 [37]	0	0.22	42	75 [48]	81	43 [60]	-32	0.02	81	53 [47]	162	41 [63]	-12	0.01
Broccoli	29	49 [45]	88	30 [31]	-19	0.16	21	65 [72]	83	33 [34]	-32	0.08	50	51 [65]	171	33 [31]	-18	0.03
Carrot	106	29 [37]	230	29 [30]	0	0.80	74	39 [35]	200	34 [25]	-5	0.64	180	31 [36]	430	31 [30]	0	0.97
Capsicum	32	15 [22]	61	23 [17]	8	0.11	26	39 [44]	55	21 [19]	-18	0.05	58	20 [30]	116	22 [18]	2	0.85
Corn	26	50 [66]	59	43 [57]	-7	0.38	10	80 [52]	56	57 [58]	-23	0.22	36	62 [62]	115	44 [56]	-18	0.27
Tomato	87	45 [33]	147	60 [53]	15	0.008	60	60 [54]	154	63 [55]	3	0.25	147	48 [54]	301	60 [55]	12	0.003
Lettuce	74	15 [21]	138	16 [16]	1	0.003	74	16 [20]	161	16 [20]	0	0.47	148	15 [21]	299	16 [20]	1	0.01
Celery/ Cucumber	40	20 [24]	108	26 [28]	6	0.47	20	24 [12]	87	30 [20]	6	0.12	60	23 [17]	195	29 [24]	6	0.13
Onion	33	18 [37]	156	13 [15]	-5	0.06	43	30 [40]	182	19 [23]	-11	0.005	76	25 [39]	338	17 [18]	-8	<0.001
Apple	99	140 [26]	245	166 [10]	26	0.003	80	140 [26]	205	166 [5]	26	<0.001	179	140 [26]	450	166 [5]	26	<0.001

Table S7: Changes to portion size (grams) between 1995 and 2007 for 13-16 year old children (cont)

	**Females**						**Males**						**Persons**					
	1995		2007				1995		2007				1995		2007			
Food	n =	Median [IQR]	n =	Median [IQR]	Δ Median	p-value	n =	Median [IQR]	n =	Median [IQR]	Δ Median	p-value	n =	Median [IQR]	n =	Median [IQR]	Δ Median	p-value
Banana	24	106 [39]	97	121 [27]	15	0.006	28	102 [36]	116	121 [27]	19	0.003	52	102 [36]	213	121 [27]	19	<0.001
Orange	26	131 [45]	158	86 [45]	-45	0.13	35	131 [51]	122	101 [45]	-30	0.08	61	131 [40]	280	99 [45]	-32	0.02
**Seasonal Fruit**																		
Grapes / Berries	13	57 [24]	69	82 [139]	25	0.24	13	86 [271]	46	60 [100]	-26	0.80	26	68 [75]	115	76 [139]	8	0.55
